# Colloidal thallium halide nanocrystals with reasonable luminescence, carrier mobility and diffusion length†
†Electronic supplementary information (ESI) available: Synthesis and additional characterization of nanocrystals, characterization of nanocrystal films, temperature-dependent phase transition, coefficient of volume expansion, PL decay dynamics, tabulated best fit parameters, and methodology analysis of ultrafast optical pump THz probe (OPTP) spectroscopy. See DOI: 10.1039/c7sc01219e



**DOI:** 10.1039/c7sc01219e

**Published:** 2017-04-19

**Authors:** Wasim J. Mir, Avinash Warankar, Ashutosh Acharya, Shyamashis Das, Pankaj Mandal, Angshuman Nag

**Affiliations:** a Department of Chemistry , Indian Institute of Science Education and Research (IISER) , Pune , 411008 , India . Email: pankaj@iiserpune.ac.in ; Email: angshuman@iiserpune.ac.in; b Centre for Energy Science , Indian Institute of Science Education and Research (IISER) , Pune , 411008 , India; c Solid State and Structural Chemistry Unit , Indian Institute of Science , Bangalore 560012 , India

## Abstract

Colloidal lead halide based perovskite nanocrystals (NCs) have been recently established as an interesting class of defect-tolerant NCs with potential for superior optoelectronic applications. The electronic band structure of thallium halides (TlX, where X = Br and I) show a strong resemblance to lead halide perovskites, where both Pb^2+^ and Tl^+^ exhibit a 6s^2^ inert pair of electrons and strong spin–orbit coupling. Although the crystal structure of TlX is not perovskite, the similarities of its electronic structure with lead halide perovskites motivated us to prepare colloidal TlX NCs. These TlX NCs exhibit a wide bandgap (>2.5 eV or <500 nm) and the potential to exhibit a reduced density of deep defect states. Optical pump terahertz (THz) probe spectroscopy with excitation fluence in the range of 0.85–5.86 × 10^13^ photons per cm^2^ on NC films shows that the TlBr NCs possess high effective carrier mobility (∼220 to 329 cm^2^ V^–1^ s^–1^), long diffusion length (∼0.77 to 0.98 μm), and reasonably high photoluminescence efficiency (∼10%). This combination of properties is remarkable compared to other wide-bandgap (>2.5 eV) semiconductor NCs, which suggests a reduction in the deep-defect states in the TlX NCs. Furthermore, the ultrafast carrier dynamics and temperature-dependent reversible structural phase transition together with its influence on the optical properties of the TlX NCs are studied.

## Abstract 2

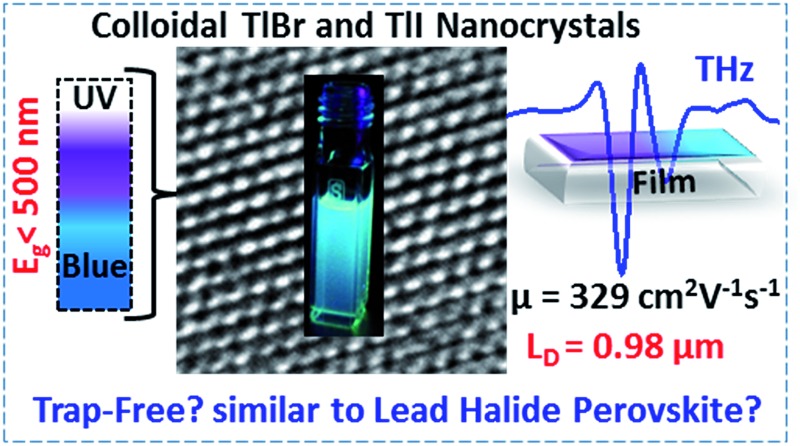
Colloidal TlI and TlBr nanocrystals are prepared, which show violet-blue luminescence, high carrier mobility and long diffusion lengths thus suggesting the suppression of deep-trap states.

## Introduction

Defect tolerance signifies the tendency of a semiconductor to retain its electronic, optical and optoelectronic properties even in the presence of defects. One of the major drawbacks of colloidal semiconductor nanocrystals (NCs) such as CdSe is surface defects, which trap charge carriers and thus severely limit their electronic, optical, and optoelectronic properties.^1^ This problem of charge trapping becomes more severe for NCs with high optical band gaps in the visible spectral region of 400 to 500 nm (3.1 to 2.5 eV). Herein, we present a new variety of colloidal semiconductor NCs in the form of TlX (X = I and Br), with optical band gaps <500 nm, which exhibit photoconductivity with high effective carrier mobilities (∼330 cm^2^ V^–1^ s^–1^), long diffusion lengths (∼1 μm), and reasonably high photoluminescence (PL) efficiency (∼10%). These results suggest the suppression of defect mediated charge trapping in TlX NCs compared to many other semiconductors with band gaps >2.5 eV.

In early 2015, Protesescu *et al.* ^2^ reported CsPbX_3_ (X = Cl, Br and I) perovskite NCs with exceptionally high (∼90% for X = Br) PL efficiency. Subsequently, we reported the suppression of PL blinking and terahertz (THz) conductivity with high carrier mobility (∼4500 cm^2^ V^–1^ s^–1^) within a CsPbBr_3_ NC.^3,4^ These results suggest that CsPbBr_3_ NCs do not exhibit mid-gap deep trap states, despite their high surface-to-volume ratio. Therefore, CsPbX_3_ NCs hold great potential as new type of practically trap-free NCs with superior optical and optoelectronic properties,^5^ as has been already reported in terms of achieving high-efficiency light emitting diodes (LED),^6,7^ solar cells,^8^ low threshold for lasing,^9^ strong nonlinear absorption together with multi-photon pumped lasing,^10,11^ and single photon emission.^12^ There has been significant progress in designing and exploring the properties of CsPbX_3_ and MAPbX_3_ (MA = methylamine) NCs with various shapes, sizes and compositions.^13–23^

**Fig. 1:**
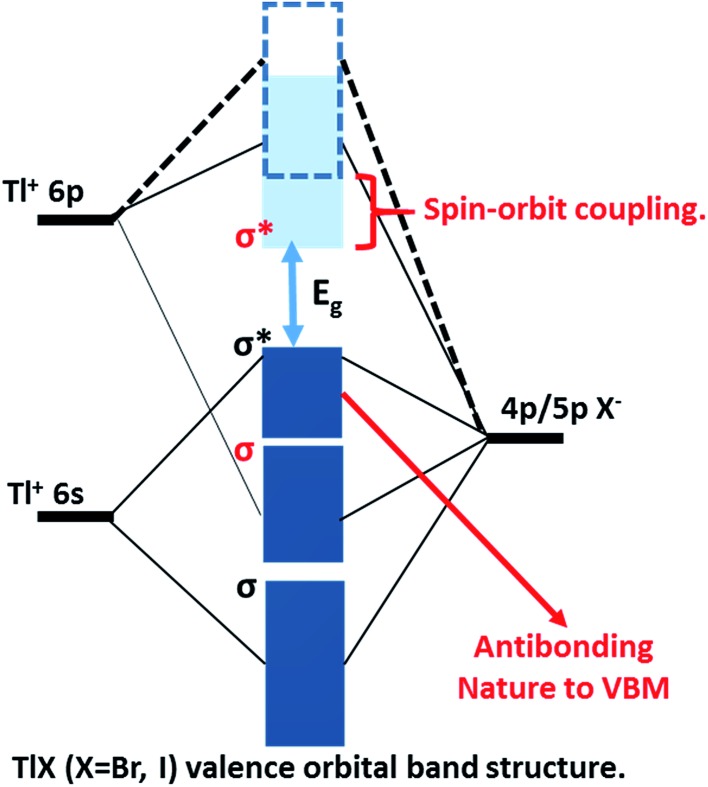
Schematic representation of the valence orbital band structure of TlX (X = Br and I) showing the bonding and antibonding orbitals forming the energy bands of the TlX solid, relative to the isolated p and s atomic orbitals of Tl^+^ and X^–^, respectively. The non-bonding Tl 6p states mainly constitute the conduction band minimum (light-blue band), which is further stabilized by strong spin–orbit coupling. The anti-bonding interactions of Tl 6s and X np constitute the valence band (dark-blue) maximum.

The successful applications of CsPbX_3_ perovskite NCs motivated us to search for other types of trap-free colloidal semiconductor NCs, particularly with high optical gaps corresponding to the UV-blue region, where the probability of the formation of deep-trap states increases. In this regard, TlX NCs have great potential since Tl^+^ possesses a number of common characteristics with Pb^2+^, such as Tl^+^ is isoelectronic with Pb^2+^ and they both possess a lone pair of outermost s-electrons. The anti-bonding interactions of the filled 6s orbital of Tl^+^ with the 4p/5p orbital of halide forms the valence band maximum (VBM), as shown in Fig. 1.^24^ On the other hand, Tl^+^ 6p orbitals mainly contribute to the conduction band minimum (CBM). Another anticipated common feature between Tl^+^ and Pb^2+^ is strong spin–orbit coupling in the 6p band.^25–27^ Thus, both 6s^2^ electrons and spin–orbit coupling yield the desired electronic band structure for TlX and CsPbX_3_, where the anti-bonding states form the VBM and spin–orbit coupling stabilizes the CBM, as depicted in Fig. 1.^28^ Such electronic band structure is expected to reduce the deep-trap states in semiconductor nanocrystals. Therefore, metal halides with different compositions and structures, but with a valence orbital band structure similar to Fig. 1 are now being explored.^28–31^

At room temperature, TlI exhibits an orthorhombic structure with a band gap of 2.86 eV (434 nm),^32^ whereas, TlBr exhibits a cubic structure with a band gap of 3.05 eV (407 nm).^33^ The crystal structures of both TlI and TlBr are different from that of ABX_3_ perovskite ((A): monovalent metal/organic ion, (B): divalent metal ion, and X: halide ion), but they are analogous to ABX_3_ in terms of the valence orbital band structure. Bulk and thin films of TlX and its derivatives have already been explored as potential candidates for room temperature X-ray and gamma-ray detection.^34–36^ Herein, we report colloidal TlX NCs, where their optical properties and ultrafast optical pump THz probe (OPTP) spectroscopy suggest the suppression of deep-defect mediated charge trapping. Furthermore, temperature dependent structural phase transition is observed using variable-temperature X-ray diffraction (XRD) and differential scanning calorimetry (DSC). To the best of our knowledge, this is the first report on colloidal TlX NCs, which show interesting properties suitable for optoelectronic applications in the UV-blue region. However, the toxicity of Tl based compounds is well-known,^37,38^ which will be a major hindrance for the many real life applications of TlX NCs.

## Results and discussion

### Synthesis

We employed a hot-injection route for the colloidal synthesis of TlX NCs, as discussed in the (ESI†). Colloidal synthesis of TlX NCs is complicated due to the highly reactive ionic nature of the reaction between Tl^+^ and X^–^ ions. The fast growth of NCs makes it difficult to isolate smaller NCs and study the growth kinetics. Another intrinsic behaviour of TlI is that the crystal phase transition occurs at around the reaction temperatures (170–300 °C). Therefore, controlling the crystal structure of TlI NCs becomes more challenging. The growth of TlX NCs during our synthesis was stopped immediately after the precursors were injected by applying a bath containing dry ice in acetone. Lowering the reaction temperature is beneficial to achieve both crystal structure homogeneity for the TlX product NCs and suppression of NC growth. The product NCs exhibit colloidal stability for about a week under ambient conditions. Further modification of the reaction methodology is required to achieve better separation of the nucleation and growth processes in order to control the size and shape of the nanocrystals over a wider range. In this regard, exploring various surface ligands and washing procedures might help to achieve better quality nanocrystals with improved colloidal stability and optical properties.

### Structure and optical properties of TlI NCs

**Fig. 2:**
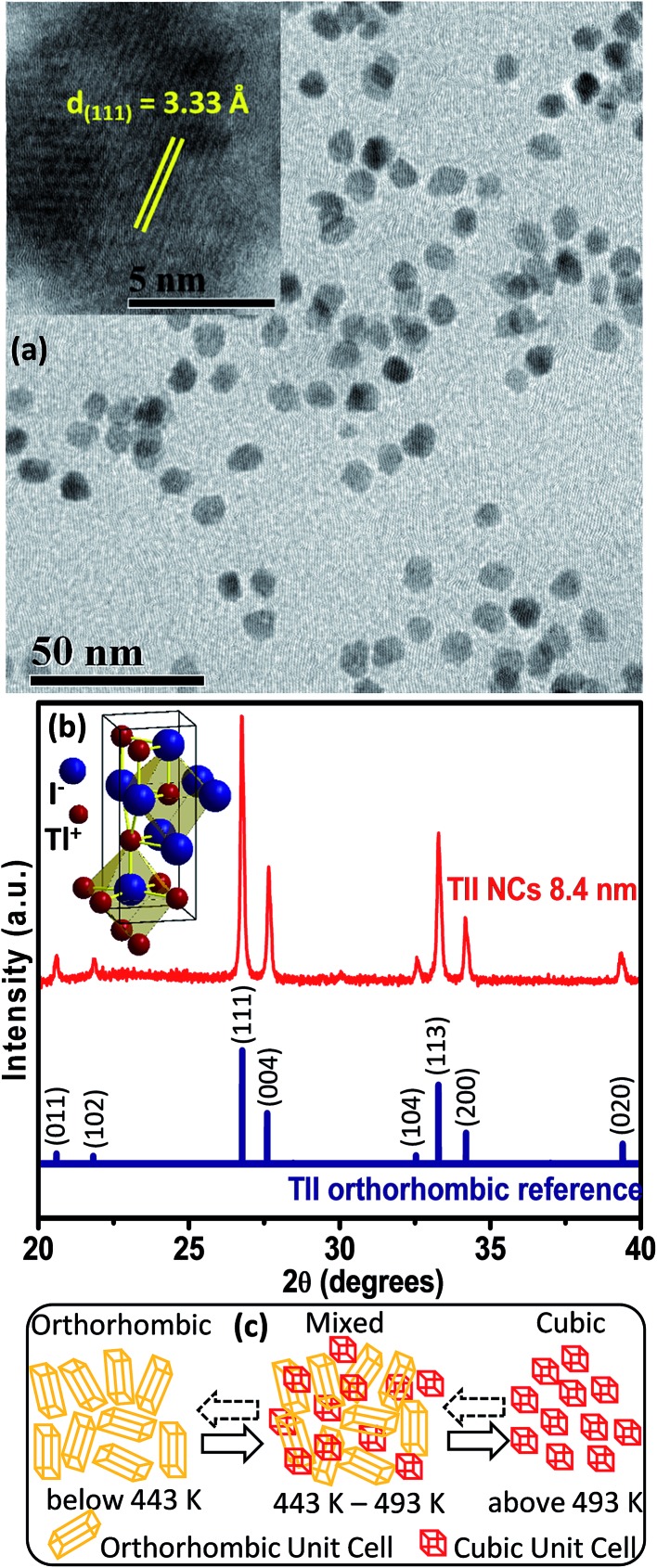
(a) TEM image of TlI NCs with an average size of 8.4 nm. The inset shows the HRTEM image of a single TlI NC displaying lattice fringes of (111) planes. (b) Comparison between the powder XRD diffraction patterns of TlI NCs (red, top) and orthorhombic bulk TlI reference (blue, bottom). The inset shows the TlI orthorhombic unit cell with coordination network of seven ions around each ion. (c) Schematic depicting the structural phase changes of TlI NCs in the given temperature range. Dashed arrows in the reverse direction indicate a relatively slower reverse transition.

Elemental analysis by energy dispersive X-ray spectroscopy (EDS), which is shown in Fig. S1 of the ESI,† and scanning electron microscopy (SEM) (Fig. S2, ESI†) validate the formation of spherical TlI NCs. The transmission electron microscopy (TEM) image shown in Fig. 2a and corresponding size distribution histogram (Fig. S3 in ESI†) show that the TlI NCs prepared at 170 °C exhibit an average diameter of 8.4 ± 1.2 nm. The inset in Fig. 2a shows the high resolution TEM (HRTEM) image of a single TlI NC with a diameter of 8.4 nm displaying (111) lattice fringes with an interplanar distance of 3.33 Å. The Fourier transform infrared (FTIR) spectra and proton nuclear magnetic resonance (^1^H NMR) spectra given in Fig. S4 of the ESI† suggest the presence of both oleate and oleyl ammonium in the product NCs.

The powder XRD pattern in Fig. 2b confirms the orthorhombic phase of the TlI NCs at room temperature, which is similar to bulk^39^ TlI. The inset in Fig. 2b shows an orthorhombic unit cell of the TlI lattice with coordination polyhedra of seven ions around each Tl^+^ and I^–^ ion. Thus the coordination environment of TlI is different from that in CsPbI_3_ cubic perovskite.^2^ Samara *et al.* reported polymorphism in bulk TlI with ∼1% difference in lattice energy between the cubic and orthorhombic phases.^39^ This prompted us to study the possible crystal phase transition in our organic capped colloidal NCs, where surface energy plays a crucial role in contrast to their bulk counterpart. The powder XRD patterns of the TlI NCs in the temperature range of 303 to 543 K together with the DSC data (given in Fig. S5 in the ESI†) yields the schematic shown in Fig. 2c. The TlI NCs (8.4 nm) exhibit an orthorhombic structure at room temperature, but transform into a cubic phase at 443 K similar to their bulk^39^ counterpart. A mixed phase is observed in the temperature range of 443–493 K. Then the NCs are cooled and the transition is monitored in the reverse direction from cubic to orthorhombic phase. However, this reverse phase-transition is observed to be a slow process. It takes many hours at 303 K before the sample completely reverts into a pure orthorhombic phase, as shown by the XRD patterns in Fig. S5a in the ESI.† Such structural phase transition from orthorhombic to cubic TlI NCs is accompanied by a change in color from pale yellow to reddish-orange (see Fig. S6 in the ESI†). Furthermore, the linear fit of the unit cell volume (*V*) as a function of temperature (*T*) for the TlI NCs is shown in (Fig. S7 in the ESI†), which results in a similar coefficient of volume expansion (*α*_*V*_) for both phases of the TlI NCs. For the orthorhombic phase, *α*_*V*_ = 1.47 × 10^–4^ K^–1^ and for the cubic phase *α*_*V*_ = 1.5 × 10^–4^ K^–1^. These *α*_v_ values are in accordance with the previously suggested values for bulk^39^ TlI.

**Fig. 3:**
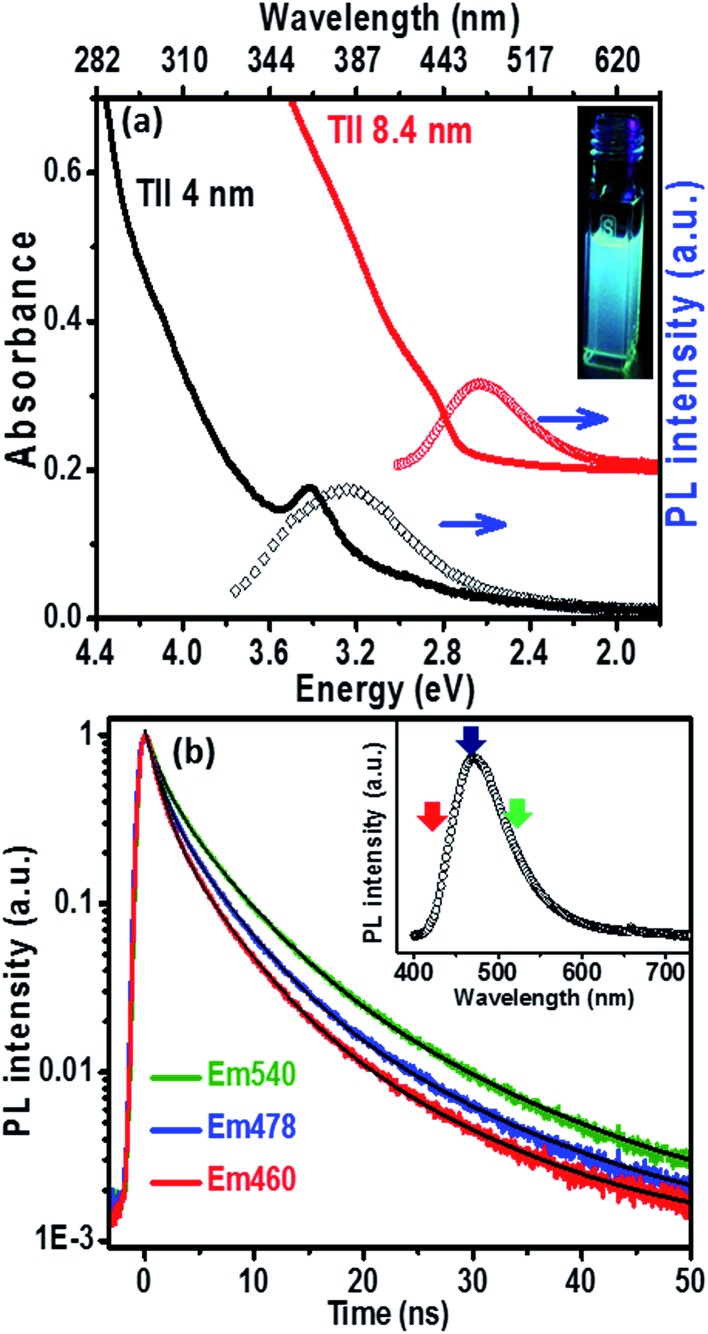
(a) UV-visible absorption and PL spectra of TlI NCs with average sizes of 8.4 nm (red spectrum) and 4 nm (black spectrum). The absorption and PL spectra (red) of the 8.4 nm sized NCs are vertically offset (by a constant 0.2) for clarity. Inset shows a photograph of the luminescence from a colloidal dispersion of the 8.4 nm TlI NCs after irradiation with 365 nm UV light. (b) PL decay profiles of the colloidal TlI NCs (8.4 nm) at three different emission wavelengths after excitation at 400 nm. Inset shows the corresponding PL plot and arrows depict the emission wavelength probed in the time resolved PL decay scan.

The UV-visible absorption spectrum (Fig. 3a) of the 8.4 nm TlI NCs (prepared at 170 °C) dispersed in hexane shows an excitonic absorption peak at 438 nm (2.83 eV) which is similar to the optical gap of bulk (2.86 eV)^32^ orthorhombic TlI. According to prior reports, the Bohr excitonic diameter of TlI NCs is expected to be in the range of 9–12 nm for the orthorhombic phase (Table S1 of ESI†).^40^ Therefore, weak confinement of charge carriers is expected for the 8.4 nm TlI NCs, which exhibit an optical gap similar to that of bulk TlI. When the TlI NCs were prepared at 200 °C, smaller NCs with an average diameter of 4 nm were obtained (see TEM image in Fig. S8 of the ESI†). The UV-visible absorption spectrum (Fig. 3a) of the 4 nm TlI NCs exhibits a sharper excitonic feature at 365 nm (3.44 eV). This is a significant blue-shift compared to the optical gap of bulk TlI, which displays the characteristic of quantum confinement in these NCs. Furthermore, in contrast to bulk or thin films of TlI, our colloidal TlI NCs exhibit excitonic features at room temperature, which suggest an increase in excitonic binding energy in the NCs because of the quantum confinement of electron and hole wavefunctions. Films made by casting the TlI NCs onto polystyrene substrates show similar optical gaps in their UV-visible absorption spectra, as shown in (Fig. S9 of the ESI†). It should be noted that because of the fast growth rate it is difficult to isolate smaller sizes of TlI NCs, thus continuous tuning of the optical gap in the range of 365 nm (3.44 eV) to 438 nm (2.83 eV) is difficult to achieve with reproducibility.

The PL peaks (Fig. 3a) centred at 382 nm and 480 nm correspond to 4 nm and 8.4 nm TlI NCs, respectively. The inset of Fig. 3a shows a photograph of the PL from the colloidal TlI NCs (8.4 nm) dispersed in hexane under UV illumination (365 nm). The PL QY of ∼6% was obtained for the 8.4 nm TlI NCs and ∼4% for the 4 nm TlI NCs. To the best of our knowledge, prior literature on bulk and thin films of TlI NCs did not report PL at room temperature. Agekyan *et al.* ^41^ reported similar PL at 80 K from microcrystals of TlI embedded in a porous matrix. Probably, the smaller size of our NCs increases the overlap between the electron and hole wavefunctions which increases the transition probability for PL. The broadening of PL in our TlI NCs suggests possible contribution from both exciton and shallow defect states in the PL.

To understand the PL mechanism, the PL decay dynamics of the TlI NCs shown in Fig. 3b were fitted with a tri-exponential decay function and the best-fit parameters are summarised in Table S2 of the ESI.† A non-radiative lifetime, *τ*_1_ ∼ 1 ns, and two radiative lifetimes, *τ*_2_ ∼ 4 ns and *τ*_3_ ∼ 12 ns, are obtained at the three different wavelengths of emission. Such short radiative lifetimes indicate transitions involving delocalized states, which might arise from both band edges (or excitonic) and shallow defect states.^42^ Unlike the mid-gap deep trap states, shallow defects are almost delocalized and optically active. The absence of long (∼100 ns) lifetimes shows the absence of deep-trap related radiative recombination in the NCs. The qualitative nature of the PL decay profiles is similar for all three emission wavelengths shown in Fig. 3b. However, a greater contribution from the non-radiative decay channels is observed at lower wavelengths probably because of the additional non-radiative pathways at higher energies including energy transfer from higher optical-gap NCs to lower optical-gap NCs within the ensemble.

### Structure and optical properties of TlBr NCs

**Fig. 4:**
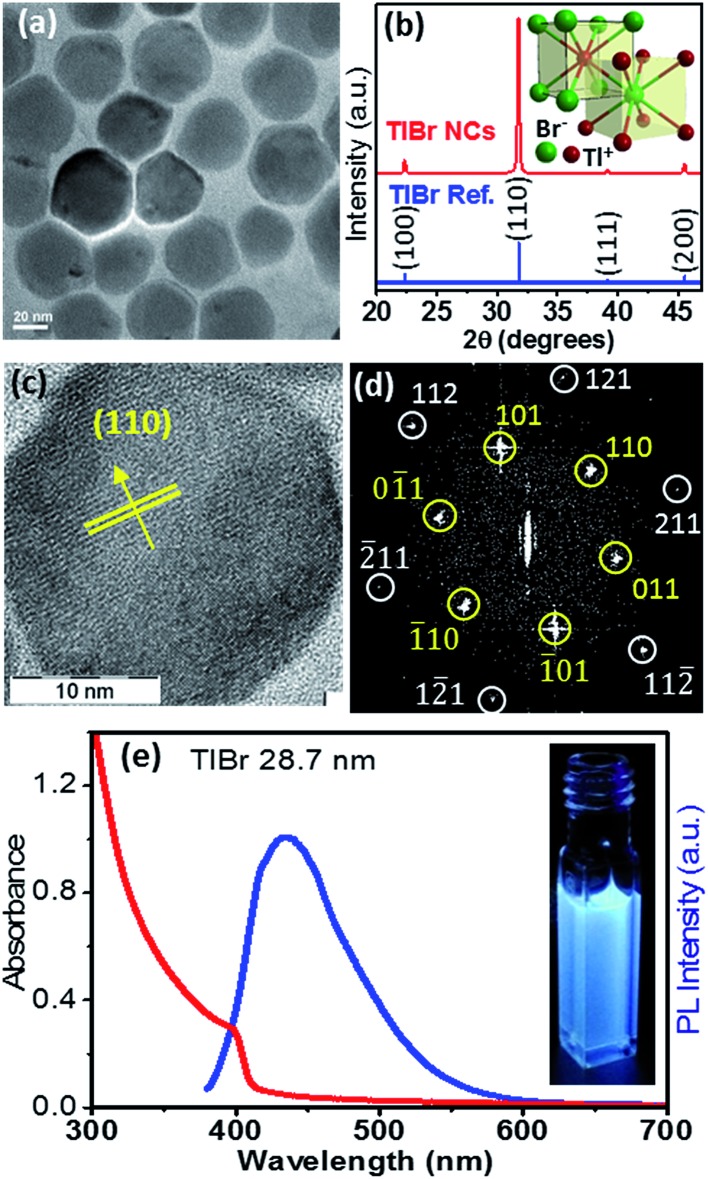
(a) TEM image of the TlBr NCs with an average diameter of 28.7 nm. (b) Powder XRD pattern of the TlBr NCs showing the cubic phase; inset shows a schematic unit cell for the cubic phase of TlBr with coordination polyhedra of 8 atoms around each ion. (c) HRTEM image of a single TlBr NC and (d) corresponding FFT pattern of the TlBr NC showing the high crystallinity of the NC. (e) UV-visible absorption and PL spectra of a colloidal dispersion of the TlBr NCs; inset shows luminescence from the TlBr NCs dispersed in hexane under 365 nm UV radiation.

In this section we discuss the results of colloidal TlBr NCs. EDS (Fig. S10 in the ESI†) and SEM (Fig. S11 in the ESI†) show that the composition of the product is TlBr and a spherical NC morphology, respectively. The TEM image in Fig. 4a (and Fig. S12 in ESI†) shows an average diameter of 28.7 ± 3.2 nm for the TlBr NCs. The powder XRD pattern of the TlBr NCs, which is displayed in Fig. 4b, shows a cubic phase, similar to bulk^43^ TlBr, at room temperature. The inset in Fig. 4b shows a schematic of the cubic unit cell of the TlBr lattice with coordination polyhedra of 8 ions around each Tl^+^ and Br^–^ ion in cubic symmetry. Furthermore, the cubic phase of the TlBr NCs is stable in the temperature range of 303 K to 573 K with *α*_*V*_ = 3.2 × 10^–5^ K^–1^ (see Fig. S13 in the ESI†). The HRTEM image of a single TlBr NC shown in Fig. 4c and the corresponding fast Fourier transform (FFT) pattern in Fig. 4d confirm the single crystalline nature of the cubic phase of the TlBr NCs. FTIR and ^1^H NMR data in Fig. S4 in the ESI† show the presence of both oleate and oleyl ammonium on the surface of the NCs, similar to the TlI NCs.

The UV-visible absorption spectrum of the TlBr NCs in Fig. 4e shows an excitonic feature at 400 nm (3.1 eV), which is in accordance with the optical gap of 3.05 eV for bulk^33^ TlBr. The diameter (28.7 nm) of the TlBr NCs is more than the estimated values (Table S1 in the ESI†) of the Bohr excitonic diameter (9.7 to 24.6 nm) for TlBr; therefore, these NCs experience a very weak or no quantum confinement effect. Films of the TlBr NCs on polystyrene substrates exhibit an optical gap similar to colloidal NCs (see Fig. S14 in the ESI†). The PL spectrum (Fig. 4e) of the TlBr NCs shows a peak at 435 nm with a QY of ∼10% at room temperature. The photograph in the inset of Fig. 4e shows the PL of the TlBr NCs dispersed in hexane after illumination with UV light (365 nm). The PL decay profiles of the colloidal TlBr NCs are qualitatively similar to that of the TlI NCs, which are shown in Fig. S15 in the ESI.†

### Carrier dynamics from OPTP spectroscopy

**Fig. 5:**
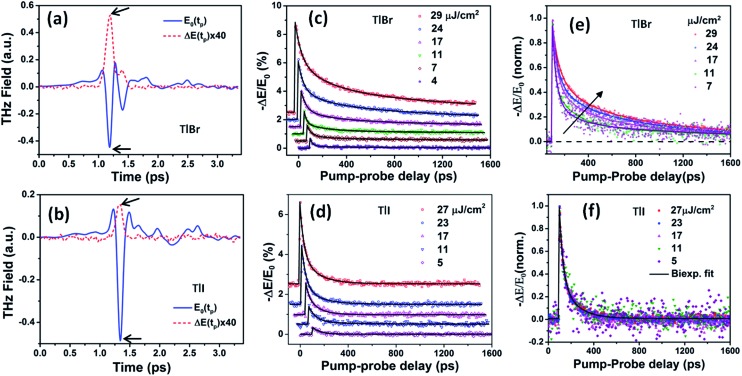
Reference (pump-off) THz waveform (blue solid line) and photo-induced change in THz transmission (red dashed line) for (a) (28.7 nm) the TlBr and (b) (8.4 nm) TlI NC films recorded at 5 ps pump-probe delay at 27 and 23 μJ cm^–2^ pump fluence, respectively. The arrows indicate the peak THz fields that were recorded to obtain the THz transients shown in panels (c) and (d). The corresponding complex conductivity spectra are shown in Fig. S17 in the ESI.† Photoinduced THz transients for (c) TlBr and (d) TlI NC films at different pump fluences. Pump wavelength used is 400 nm. Data at higher fluences are offset for visual clarity. The solid lines are multiexponential fits of the experimental THz transients (points). THz transients normalized to the peak values for (e) TlBr and (f) TlI.

**Fig. 6:**
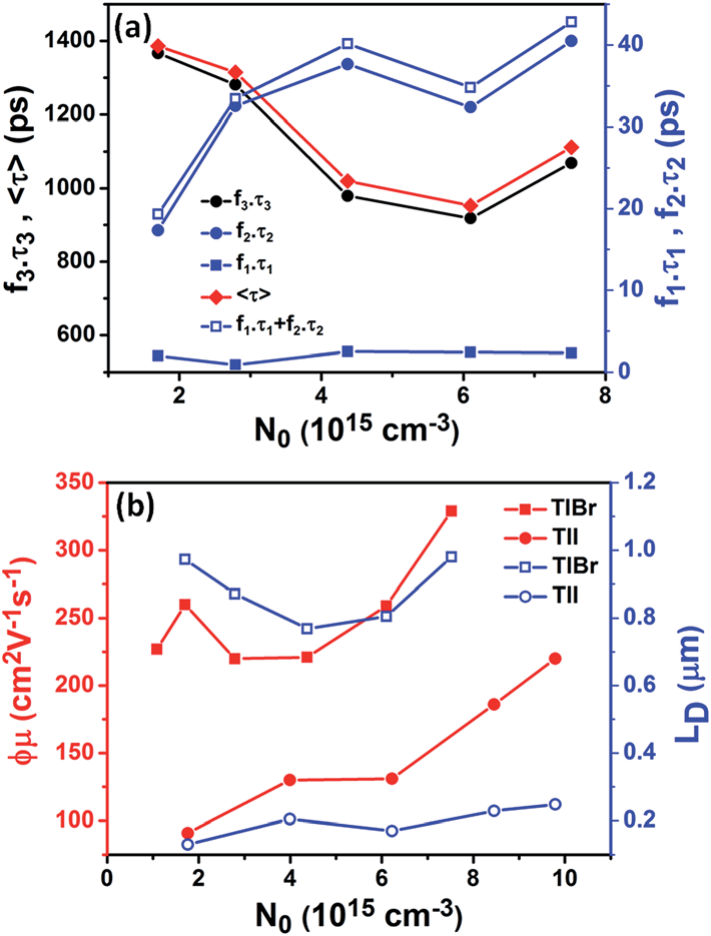
(a) The contribution of different recombination channels (*f*_*i*_ *τ*_*i*_) in the case of the TlBr NCs is plotted against initial carrier density (twice the density of photons absorbed). (b) Effective carrier mobility (red) and carrier diffusion length (blue) for the (28.7 nm) TlBr and (8.4 nm) TlI NCs, as a function of initial carrier density (fluence).

We studied films of the TlBr and TlI NCs with average diameters of 28.7 nm and 8.4 nm, respectively. The NCs were coated on polystyrene substrates for OPTP measurements. The pump-induced change in THz transmissions were recorded by measuring the peak THz field (as shown by arrows in Fig. 5a and b) as the pump-probe delay was varied up to 1.5 ns (see Fig. 5c and d). The photoconductivity (Δ*σ*(*t*_p_)) of NC films on a non-conducting substrate (polystyrene in the present work) is proportional to the photo-induced THz transmission (–Δ*E*(*t*_p_)/*E*_0_(*t*_p_)), which is given by the following equation,^44^
1
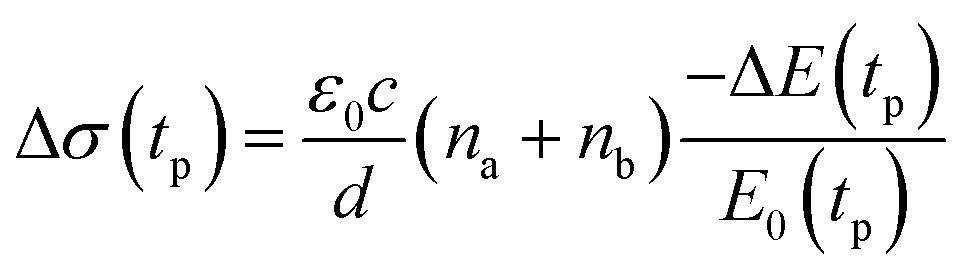
where, *ε*_0_ is the permittivity of free space, *c* is the speed of light in vacuum, *d* is the sample thickness, and *n*_a_ and *n*_b_ are the refractive indices of the media on both sides of the film. In our experiment, *n*_a_ = 1, *n*_b_ = 1.525 are the refractive indices of air and the polystyrene substrate, respectively. The thickness of the TlBr and TlI NC films are 11.2 μm and 8.1 μm, respectively, as obtained from the optical surface profilometry scans shown in Fig. S16 in the ESI.† Photoconductivity depends on the product of carrier mobility (μ) and charge carrier density (*N*_0_). Based on the previous reports^4,45^ on metal halide perovskites, we assume that the carrier mobility does not change significantly within the temporal window of our experiments. Thus, the THz transients essentially represent the temporal evolution of the free carrier density. Here, we argue that the THz probe responds only to the free carriers, not to the bound electron–hole pairs (excitons).^46^ This is evident from the positive real conductivities observed in the complex conductivity spectra shown in Fig. S17 in the ESI.† However, we do not expect to observe Drude-like behaviour because of the possible charge localization due to disorder in the medium. The THz transients measured at different fluences are shown in Fig. 6c and d for TlBr and TlI, respectively. The photoconductivities reach their peak values instantaneously (within the instrument response time of ∼300 fs) upon the arrival of the pump light for both films, which indicates the generation of free electrons and holes immediately after excitation. As expected, the peak photoconductivity increases with an increase in pump fluence for both samples. The decay profiles are influenced by the recombination dynamics of the free carriers, which seem to be significantly different for TlBr and TlI. The dynamics are much slower in case of TlBr than that in TlI. To unravel the carrier recombination mechanisms we fitted the THz transients to a multiexponential function convoluted with a Gaussian function of the form:^47^
2
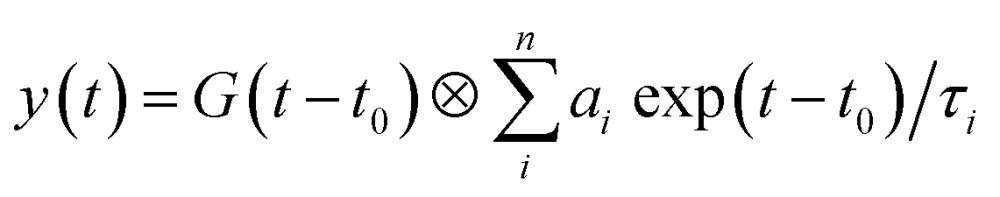
where, *a*_*i*_ is the contribution of the *i*th process with a time constant *τ*_*i*_ towards the overall dynamics. The fits are shown as solid lines in Fig. 6c and d. Three exponentials are required to obtain a good fit of the THz transients to eqn (2) at all fluences for the TlBr NCs; whereas only two exponentials are sufficient enough for the TlI NCs at all fluences except 5 μJ cm^–2^. In this case, a single exponential with a time constant of 71.4 ps could fit the decay profile reasonably well. The details of the fits are given in Tables S3 and S4 in ESI.† In the case of the TlBr NCs, at different fluences the time constants of the fastest recombination process range from 22 to 51 ps and the slowest process has time constants in the range of 1.13 to 1.66 ns. The intermediate process increases from 156 to 254 ps with an increase in fluence. On other hand, in case of the TlI NCs, the faster process has a timescale between 22 and 35 ps, whereas the slower process shows time constants ranging from 71 to 147 ps at different pump intensities. The average life times (∼1 ns and higher) for TlBr are about an order of magnitude longer compared to that for TlI (∼100 ps). The average lifetimes (*τ*) are calculated as *τ* = ∑*f*_*i*_ *τ*_*i*_, where, *f*_*i*_ (*f*_*i*_ = *a*_*i*_ *τ*_*i*_/∑*a*_*j*_ *τ*_*j*_) signifies the contribution of the *i*th process towards the entire recombination dynamics.^4,48^

To compare the fluence dependent carrier dynamics of the TlBr NCs and TlI NCs, we normalized the THz transients to their maximum values, which are shown in Fig. 6e and f. The free carrier recombination dynamics in the TlBr NCs is strongly fluence (carrier density) dependent (Fig. 5e), whereas for the TlI NCs it is completely fluence independent (Fig. 5f). In the case of the TlI NCs, we attribute the faster process to charge trapping and the relatively slower process to electron–hole recombination. The recombination dynamics in the TlBr NCs are quite complex and interesting. As shown in Fig. 5e, the recombination rate seems to be independent of fluence in the very early stage (few tens of picoseconds). However, with an increase in fluence the recombination rate slows down in the intermediate time scale (50 to 300 ps), but becomes faster at a later stage (>300 ps). Overall, the average lifetime reduces with an increase in fluence.

To understand how different components of the recombination mechanism respond to the change in pump fluence, we plotted the contribution of individual processes (*f*_*i*_ *τ*_*i*_) towards the overall decay (*τ*) as a function of fluence in Fig. 6a. The contribution of the fastest process (*f*_1_ *τ*_1_) is mostly independent of fluence. However, the contribution of the second process with intermediate time constants (*f*_2_ *τ*_2_) increases with an increase in fluence (becomes slower), whereas the slowest process (*f*_3_ *τ*_3_) becomes faster. The overall dynamics represented by the average life time (*τ*) becomes faster with an increase in carrier density, which follows the trend of *f*_3_ *τ*_3_. We assign *τ*_1_ to the carrier trapping process (monomolecular or single particle) which is independent of pump fluence, and *τ*_3_ to the bimolecular (two particle) electron–hole recombination. The process with the time constant *τ*_2_ could possibly be a different type of carrier trapping that becomes slower with an increase in pump fluence because of the reduction in the density of trap states. A possible kinetic scheme for this process is: 
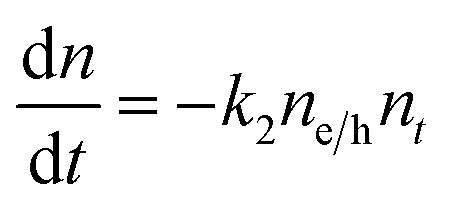
, where, *n*_e/h_ is the carrier density and *n*_*t*_ is the density of trap states which does not become saturated even at the highest pump fluence used here. This process is different from the other carrier trapping (*τ*_1_), already mentioned for the TlBr NCs and TlI NCs, where the trap states are saturated even at the lowest fluence of this study. Alternatively, the *τ*_2_ process could be an Auger recombination that leads to electronic heating, which slows the system down with an increase in fluence.^49^ Clearly, the recombination mechanism depends strongly on the excitation fluence within our experimental range. Measuring carrier dynamics at fluences lower than our experimental range will perhaps provide more insight into the carrier recombination mechanism. Certainly, further studies are required to understand the recombination mechanism, which will be investigated in our future effort.

### Mobility and diffusion length

The initial effective mobility values were calculated from the peak photoconductivities (Δ*σ*) for both samples at different pump fluences. The effective carrier mobilities were calculated using equation
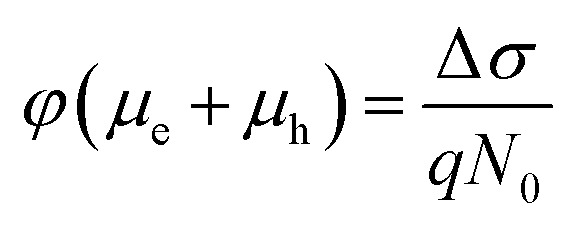
, where, *q* is the elementary charge and *N*_0_ is the total carrier density. *N*_0_ = 2*N*_ph_ *φ*, where, *N*_ph_ is the density of absorbed photons and *φ* is the photon to free carrier conversion ratio. Our approach evaluates the total carrier mobility (*μ*) which has contribution from both electron mobility (*μ*_e_) and hole mobility (*μ*_h_). Here, we assume that each absorbed photon is converted into two charge carriers (electron and hole). Depending on the pump fluence, we find that the initial carrier density is in the range of 1.1 × 10^15^ cm^–3^ to 7.5 × 10^15^ cm^–3^ for the TlBr NCs and 1.8 × 10^15^ cm^–3^ to 9.8 × 10^15^ cm^–3^ for the TlI NCs. We emphasize that these carrier densities (pump fluences) are quite modest for evaluating mobilities and carrier recombination dynamics.^45,46^ Since the actual value of *φ* is not known, these measurements yield effective initial mobilities (*φμ*). It should be noted that this analysis considers the upper limit of *N*_ph_ since we ignore the reflection and scattering losses if any. The initial mobilities (see Fig. 6b) vary from 220 to 329 cm^2^ V^–1^ s^–1^ in the case of the TlBr NC film and from 91 to 220 cm^2^ V^–1^ s^–1^ in the case of the TlI NC film at different fluences in the range of 0.85–5.86 × 10^13^ photons per cm^2^. However, the *φμ* values are quite similar at lower pump intensities. Further improvement in the estimation of the carrier mobility values may be achieved by using a lower excitation fluence than that of our experimental range. The mobility values obtained here are reasonably high compared to that of many other NC systems, as discussed later.

Next, we calculated the carrier diffusion length, 
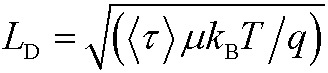
; where, *τ* is the average carrier life time, *μ* is the mobility, *k*_B_ is the Boltzmann constant, *T* is temperature, and *q* is the elementary charge. The diffusion lengths and effective mobilities obtained for the TlBr and TlI NCs at different fluences are shown in Fig. 6b and summarised in Table 1. As expected, the mobility and the diffusion length have very similar dependence on carrier density (fluence) for a particular sample. However, the trends are quite different for the TlBr and TlI NCs. At a particular carrier density the mobility for the TlBr NCs is roughly twice that of the TlI NCs. On the contrary, the diffusion lengths in the TlBr NCs are almost an order of magnitude longer than that of the TlI NCs since the average lifetimes of the TlBr NCs are an order of magnitude higher. At certain fluences, the *L*_D_ values for the TlBr NCs are in microns with mobilities of ∼300 cm^2^ V^–1^ s^–1^, which is beneficial for various optoelectronics such as UV, X-ray, gamma-ray detectors. However, one should be careful in interpreting the mobility and diffusion length values obtained from this study and should not expect them to be same in real devices composed of these NCs. OPTP probes the local AC photoconductivity and hence does not reflect any long range disorder in the system.

**Table 1:** Extracted parameters at different pump fluences. Excitation fluence is given in energy per cm^2^ per pulse (column 1) as well as no. of photons per cm^2^ per pulse (column 2). Carrier density, *N*_0_ = 2*N*_ph_ *φ*, where, *N*_ph_ is the density of the absorbed photons and *φ* is the photon to carrier conversion ratio. Pump wavelength is 400 nm

Fluence (μJ cm^–2^)	Photons (10^13^ cm^–2^ per pulse)	*N*_0_ (10^15^ cm^–3^)	Peak conductivity (S m^–1^)	Mobility (cm^2^ V^–1^ s^–1^)	Avg. lifetime (ps)	Diffusion length (μm)
**TlBr NCs**						
29	5.86	7.52	39.7	329	1112	0.981
24	4.76	6.10	25.3	259	953	0.805
17	3.41	4.37	15.4	221	1020	0.769
11	2.18	2.79	9.9	220	1315	0.872
7	1.33	1.70	7.1	260	1386	0.973
4	0.85	1.09	4.0	227		

**TlI NCs**						
27	5.38	9.79	34.6	220	106.2	0.248
23	4.64	8.45	25.1	186	107.6	0.229
17	3.42	6.22	13.0	131	84.8	0.171
11	2.19	3.99	8.3	130	123.4	0.205
5	0.97	1.76	2.6	91	71.4	0.130

### Comparison of TlX NCs with other semiconductor NCs with optical band gaps >2.48 eV (<500 nm)

As the optical band gap increases the probability of finding a defect state deep within the gap increases. For NCs, this problem becomes more severe because of the abundance of surface defects owing to their large surface-to-volume ratios. Thereby, the development of semiconductor NCs with reduced deep-defect related trap-states and optoelectronic properties in the UV to blue region of the electromagnetic spectrum is highly challenging. This troublesome issue of deep trap-states is often reflected in the poor luminescence efficiency, low carrier mobility and small diffusion lengths of charge carriers.

Table 2 suggests that the 10% PL QY of our TlX NCs is indeed a reasonably high number in the UV-blue region. The PL QY may be further improved by synthesizing core/shell NCs with type-I band alignment at the interface;^66^ however, such a structure is often detrimental for charge transport. It should be noted that bulk TlX is known to have higher dielectric constants (*ε*_r_) (*ε*_r_ = 30.6 for TlBr and *ε*_r_ = 21.6 for TlI at 20 °C)^67^ compared to materials such as CdS (*ε*_r_ = 8.9), CdSe (*ε*_r_ = 10.2), and ZnSe (*ε*_r_ = 9.2). This high dielectric constant in TlX is expected to promote excitonic dissociation which is undesirable for PL, but desirable for photoconductivity. A high dielectric constant not only reinforces decoupling of photo-generated excitons, but also screens charge carriers from ionic trap-states thereby improving charge transport.^28^

**Table 2:** Comparison of the PL QY, carrier mobility and carrier diffusion length of the TlX NCs in this study with various well-studied semiconductor NCs that exhibit optical gaps <500 nm^*a*^

Sample	Optical gap (eV), (nm)	PL QY (%)	Mobility (cm^2^ V^–1^ s^–1^)	Diffusion length (μm)	Method adapted to measure mobility
TlBr NCs (this work)	3.1, 400	10	329	0.98	THz
TlI NCs (this work)	2.8, 436	6	220	0.25	THz
TlBr bulk^50,51^	—	—	*μ*_e_ = 40.2, *μ*_h_ = 11.8		Conductivity, drift-mobility
CsPbCl_3_ NCs^52^	3.02, 410	3, 1*	—	—	
Carbon-dots QDs^53–55^	2.69, 460	4–10, 57*	*μ_e_ = 8.5 × 10^–5^, *μ_h_ = 3.8 × 10^–5^	—	FET
CdS nanorod^56,57^	3.2, 383	10	32	—	*I*–*V*
CdS NCs^58,59^	2.58, 480	6.4	—	—	
CdS thin film bulk^60^	—	—	17	—	TFT
CdSe NCs^47,61^	2.4–2.7, 450–500	1–10	1–100	—	THz
ZnSe NCs^62^	3.3–2.8, 370–430	10–50	—	—	
ZnSe single crystal^63,64^			267, 40*, 0.017*		THz, *I*–*V*
CdSe/ZnS core shell^65^	2.53, 490	30	—	—	

^*a*^Asterisk (*) denotes data obtained from film.

We measured the effective carrier mobility and diffusion length using contact-less THz AC photoconductivity with excitation fluence in the range of 0.85–5.86 × 10^13^ photons per cm^2^, similarly to ref. 40. In the absence of available surface engineering methodologies for TlX NCs, this contactless method is perhaps the only way to measure carrier mobility. A variety of other techniques are also used for measuring carrier mobility and diffusion length in NC solids, thus there will be differences in the obtained numbers using different methodologies. In fact, even within THz spectroscopy, the numbers may be different based on whether the NC is measured in a colloidal dispersion or in film. Our measurements were done on NC films, which are closer to the architecture of future optoelectronic devices. Despite the involved uncertainties, Table 2 clearly shows that the carrier mobility and diffusion lengths of our TlX NC films are one of the highest. This combination of high PL efficiency, high carrier mobility and long diffusion lengths in a high band gap semiconductor suggests a minimal density of deep-trap states in our TlX NCs. As discussed earlier, the electron band structure in Fig. 1 indeed suggests the possibility of achieving nearly deep-trap-free TlX NCs, and our experimental results indirectly agree with that prediction. However, more direct experimental and theoretical studies are required to measure the density of deep-defect states in the TlX NCs before proclaiming them defect-tolerant NCs. These optical properties suggest that TlX NCs can be potential candidates for new solution processed semiconductors for optoelectronic application in the UV-blue region. Furthermore, they can be considered for application in solution processed materials for X-ray and gamma-ray detection; however the toxicity of Tl is a concern for any real life applications.

## Conclusions

We reported herein, colloidal TlBr (28.7 nm diameter) and TlI NCs (8.4 nm and 4 nm diameters) as new wide-bandgap materials with interesting optical properties in the near-UV to blue spectral region. The TlI NCs display an orthorhombic structure at room temperature and then undergo a reversible structural phase transition at 443 K (170 °C). On the other hand, the TlBr NCs exhibit a stable cubic phase in the studied temperature range of 303 K to 573 K. Both types of NCs exhibit excitonic absorption features and PL with a reasonably high QY of 6–10% at room temperature in the violet-blue region, in contrast to their bulk counterpart. Ultrafast OPTP spectroscopy was employed to study the carrier dynamics of the TlX NC films at different pump fluences. The TlBr NCs exhibit fluence dependent carrier dynamics which are different from the TlI NCs. Subsequently, this THz photoconductivity reveals high effective carrier mobilities of 329 and 220 cm^2^ V^–1^ s^–1^ and long diffusion lengths of 0.98 and 0.25 μm for the TlBr and TlI NCs, respectively. In general, wide bandgap (UV-blue region) semiconductor NCs often exhibit a high density of deep-defect states trapping the charge carriers. On the contrary, the combination of reasonably high PL QY, high carrier mobility and long diffusion lengths suggest a decrease in deep-defect states in our TlX NCs. These results suggest the possibility that the TlX NCs possess a defect-tolerant nature owing to their interesting electronic band structure (Fig. 1), which is similar to lead halide perovskite NCs. However, further improvement in NC synthesis and optical properties, and also computational studies are required to verify the defect-tolerant nature of the TlX NCs.
