# Supplementary material for: Colloidal thallium halide nanocrystals with reasonable luminescence, carrier mobility and diffusion length
Source: Chem Sci. 2017 Apr 19;8(6):4602–11. doi: 10.1039/c7sc01219e (PMC5618336; doi:10.1039/c7sc01219e)
Supplement: Supplementary file 1 [file SC-008-C7SC01219E-s001.pdf]

Electronic Supplementary Material (ESI) for Chemical Science.
This journal is © The Royal Society of Chemistry 2017

Electronic Supporting information (ESI)

Colloidal Thallium Halide Nanocrystals with Reasonable Luminescence, Carrier Mobility and Diffusion Length

Wasim J. Mir,[a] Avinash Warankar,[a] Ashutosh Acharya,[a] Shyamashis Das,[c] Pankaj Mandal,*[,a] Angshuman Nag*[,a]

[a]Department of Chemistry, and [b]Centre for Energy Science, Indian Institute of Science Education and Research (IISER), Pune, 411008, India.

[c]Solid State and Structural Chemistry Unit, Indian Institute of Science, Bangalore 560012, India

* e-mail: PM: pankaj@iiserpune.ac.in ; AN: angshuman@iiserpune.ac.in

Experimental Section:

Chemicals: Thallium (I) nitrate ($TlNO_3$, 99.9%), tetrabutylammonium iodide (TBAI, $\geq$ 99%), tetrabutylammonium bromide (TBABr, $\geq$ 98%), 1-octadecene (ODE, tech. 90%), oleylamine (OLA, tech. 70%), oleic acid (OA, tech. 90%), potassium bromide (KBr, $\geq$ 99%) and fluorescein sodium salt (98.5-100.5%) were purchased from Sigma-Aldrich. Hexane (98%), toluene (99.5%), 1-butanol (99.5%) and acetonitrile (99.8%) were purchased from local vendors.

Synthesis of TlI Nanocrystals (NCs): 0.5 mmol of $TlNO_3$, 15 mL 1-octadecene, 1 mL oleic acid and 1 mL oleylamine were taken in a three-neck round bottom flask and degassed at 100

^{o}C for about 1 hour. The temperature of reaction mixture was then raised to 300 ^{o}C under N_2 atmosphere till solution became clear. The temperature of solution was then lowered to 200 ^{o}C (or 170 ^{o}C). Simultaneously, in another round bottom flask, 1 mmol of tetra butyl ammonium iodide (TBAI) was mixed with 5 mL 1-octadecene and 5 mL oleylamine and degassed at 140 ^{o}C for about 90 minutes. The temperature of the iodide mixture was then raised to 200 ^{o}C under N_2 atmosphere, till the solution became clear. The temperature was then lowered to 60 ^{o}C. TlI NCs were finally obtained by swiftly injecting this iodide solution to the thallium solution maintained at 170 ^{o}C for 8.4 nm NCs (or at 200 ^{o}C for 4 nm NCs). The reaction was quenched immediately using a bath containing dry ice in acetone. The obtained reaction product was centrifuged at 3000 rpm for 1 minute and the supernatant was taken up for further washing after discarding the precipitate. Small amount (1-2 mL) of tert-butanol (or acetonitrile) anti-solvent was added to the supernatant solution to precipitate out the NCs, along with centrifugation at ~6000 rpm. The obtained precipitate was dispersed in hexane and placed in refrigerator for half an hour, following by decanting the supernatant solution after discarding any precipitate that might have formed. This colloidal NC was used for optical studies in solution phase. Excess amount of the anti-solvent starts degrading the nanocrystals. Sometimes, the washing method was repeated for a second time to get rid of any remaining oily residue, but further repetition of washing process destabilize the colloidal nanocrystals. It must be noted that thallium and its salts, similar to lead salts, are toxic in nature. Hence, necessary precautions need to be taken while using any thallium containing substance.

Synthesis of TlBr NCs: TlBr NCs were synthesized using a protocol similar to that of TlI NCs, but with some differences, which are mentioned here. 1 mmol of $TlNO_3$ and 2 mmol of tetra butyl ammonium bromide were used, and the final reaction was carried out at 300 ^{o}C.

Morphology, Structure and Optical Characterization: TEM and HRTEM images were captured on a UHR FEG-TEM, JEOL JEM-2100F electron microscope using a 200 kV electron source. SEM imaging and EDS was performed by using Zeiss Ultra Plus SEM instrument. Powder XRD data were recorded using Bruker D8 Advance X-ray diffractometer equipped with Cu K_{α} (1.54 Å) radiation. Powder XRD data at different temperatures were recorded using program controller setup in the range of 303 K to 543 K for both heating and cooling cycle. For such measurements, powder sample was placed on a platinum foil, and heated (or cooled) with a rate of 0.5 ^{o}C/min, along with a stabilization time of 5 minutes at each temperature. Throughout the experiment, the sample chamber was kept under vacuum of 10^{-3} mbar. UV-visible absorption spectra were collected using Perkin Elmer Lambda-45 UV/vis spectrometer. PL spectra and PL decay dynamics were measured using Edinburg FLS980 spectrophotometer. The PL decay dynamics were recorded using 340 nm and 400 nm nanosecond pulse LED lasers as excitation sources. DSC curve was obtained by using TA Q20 DSC instrument. FTIR studies were carried out using NICOLET 6700 FTIR spectrometer by making a pellet of TlX NC powder in KBr host. ^{1}H NMR studies of colloidal dispersion in deuterated chloroform were performed on Bruker 400 MHz NMR spectrometer. PL quantum yield (PL QY) of TlX NCs were obtained using sodium salt of fluorescein dye in water as reference (PL QY = 0.92 in water),), whereas PL QY of only 4 nm TlI NCs was obtained using quinine sulphate in 0.5 M HCl as reference (PL QY = 0.55).

Film Fabrication and Characterization: Films of TlX NCs were fabricated by drop casting colloidal dispersion of TlX NCs in hexane onto polystyrene substrate. Around 6 mg of TlX NCs were dispersed in 100 μL hexane and this concentrated dispersion was drop casted on polystyrene substrate, followed by spinning at 200 rpm and drying for 2 minutes over a spin coater. The procedure was repeated 2-3 times to obtain a film thickness around ~10 μm. The films were then kept in desiccator under vacuum drying for about 6 hours. The thickness

measurements of films were carried out by using optical surface profilometer from ZETA instruments.

Optical Pump Terahertz Probe (OPTP) spectroscopy: This is a non-contact way of measuring photo-conducting carrier dynamics in semiconductor NCs.[1-2] In a typical OPTP experiment an optical pump pulse having energy higher than the band gap excites the NCs to create charge carriers (electron in conduction band and holes in the valence band).[2] The pump-induced photoconductivity is measured by a THz probe pulse arriving at a particular delay with respect to the pump pulse. The temporal evolution of the pump induced photoconductivity is recorded by varying the pump-probe delay. Our OPTP set up is based on an ultrafast amplified laser system having 800 nm central wavelength, 4 mJ pulse energy, 1 kHz repetition rate, and ~50 fs pulse width. We generate broadband THz light from air plasma, and employ air biased coherent detection (ABCD) technique for detection.[3-4] Double lock-in technique is used to record the photoinduced THz transmission ($\Delta E(t_p)$) and the reference signal ($E_0(t_p)$) simultaneously.[5] More details of the experimental set up are given elsewhere (see ref. 4 in main manuscript). Both the TlX NC samples were excited with 400 nm pump pulse with fluences ranging from 4 to 29 $\mu J/cm^2$. All measurements were carried out at room temperature.

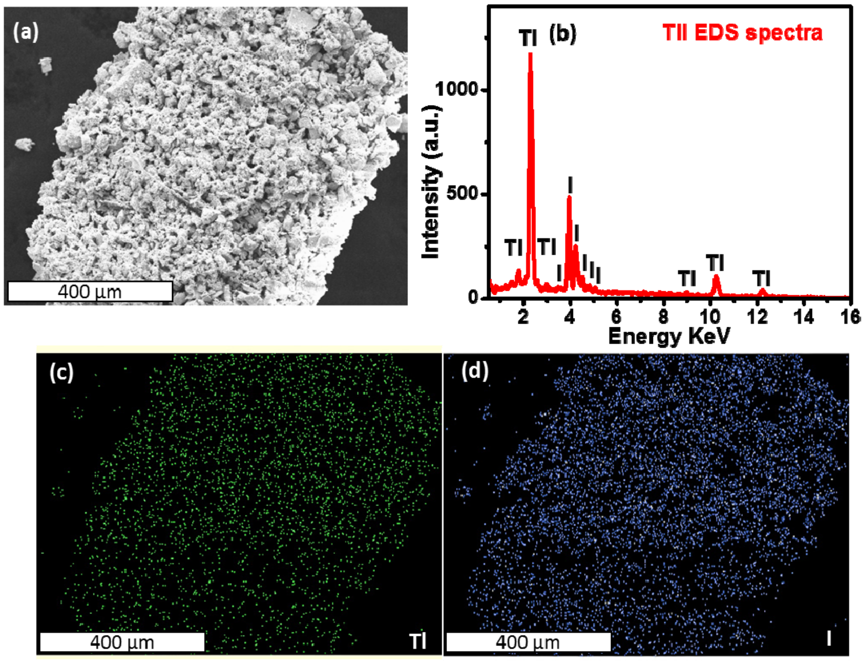

Figure S1: (a) SEM image of TlI NCs. (b) Energy dispersive X-ray spectroscopy (EDS) data acquired from the part of sample shown in Figure S1(a). Estimated Tl:I atomic ratio is 1:1. Elemental mapping of (c) 'Tl' and (d) 'I' showing homogenous distribution of both elements throughout the sample.

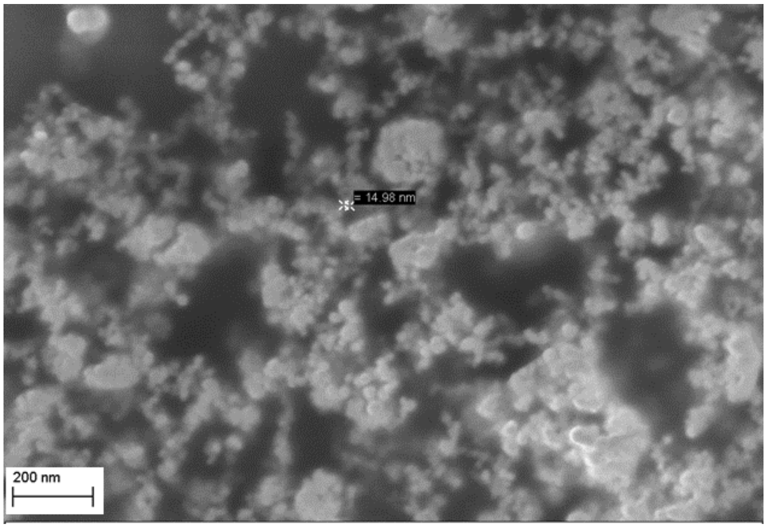

Figure S2: SEM image of TlI nanocrystals displaying spherical NCs.

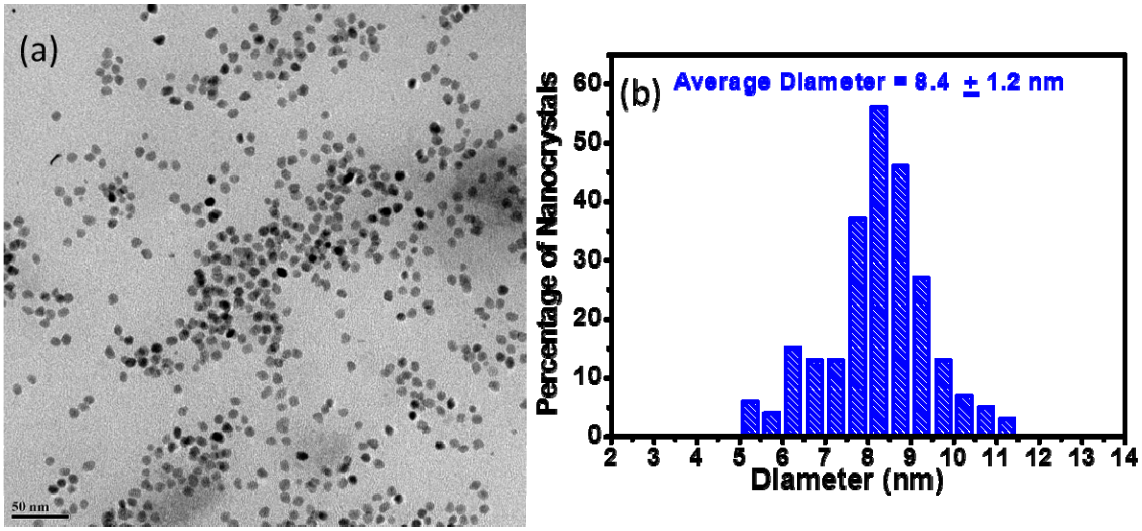

Figure S3: (a) TEM image and (b) size distribution histogram of TlI NCs.

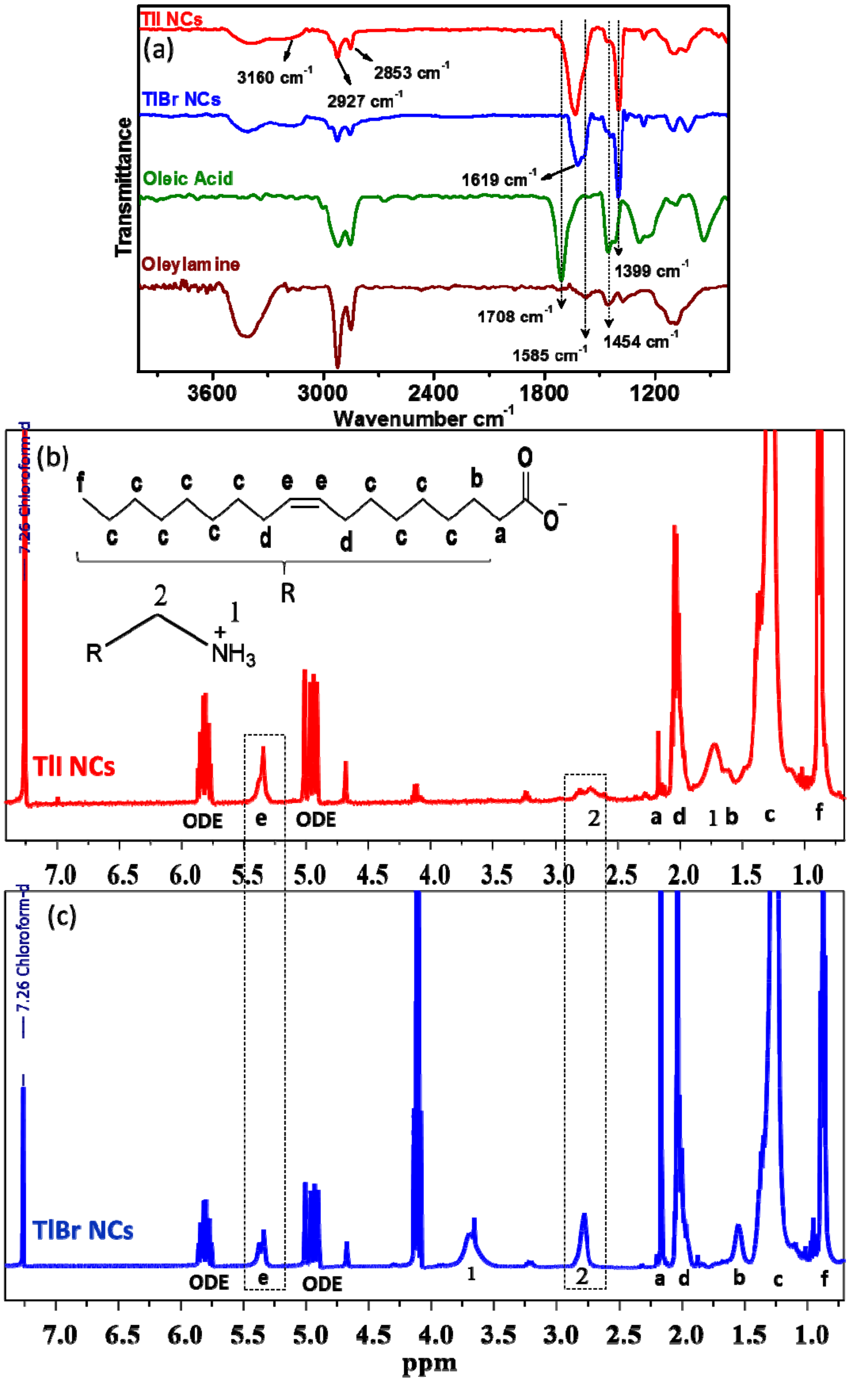

Figure S4: Surface characterization of TlI and TlBr NCs from solid state FTIR and solution phase ^{1}H NMR spectroscopy suggest binding of both oleate and oleyl ammonium on the surface of NCs. (a) FTIR spectra of TlI (red spectrum) and TlBr (blue spectrum) NCs in comparison with free oleic acid (green spectrum) and free oleylamine (wine red spectrum). Characteristic carbonyl stretching frequency of COOH functional group at 1708 cm^{-1} present in oleic acid spectra disappears in our TlX NCs and characteristic symmetric and asymmetric stretching frequency of carboxylate COO^{-} group of oleate appears at 1585 cm^{-1}. Characteristic N-H stretching frequency of oleyl ammonium appears at 3160 cm^{-1}. Also, N-H

scissor bending characteristic frequency of oleyl ammonium appears at 1619 cm^{-1} in our TlX NCs and evolves in overlap with carboxylate stretching frequency. All other frequencies observed in TlX NCs at 1399 cm^{-1}, 1454 cm^{-1}, 2853 cm^{-1} and 2927 cm^{-1} originate from hydrocarbon chain of both oleate and oleyl ammonium ligands. Solution phase ^{1}H NMR spectra ($CDCl_3$, 298K, 400 MHz) of (b) TlI and (c) TlBr NCs. Broad doublet peak from alkene protons (marked as "e") suggest binding of oleate and/or oleyl ammonium on the surface of NCs. Peaks for **a** and **b** protons of oleate suggest the binding of oleate at surface of TlI and TlBr NCs. Peaks for **1** and **2** protons of oleyl ammonium reveal binding of oleyl ammonium to the surface of NCs. The hydrogen bonded protons of oleyl ammonim attached to TlI NCs appear at smaller δ ~1.7 ppm due to lower concentration of TlI NCs in $CDCl_3$ compared to TlBr NCs in same solvent. The unassigned sharp peaks are from solvent chloroform, octadecene (ODE) and acetone.

Structural phase transition of TlI NCs. Figure S5a shows that TlI NCs (8.4 nm) initially exhibit orthorhombic structure at 303 K, and remain so when temperature is raised till 423 K (150 ^{o}C). At 433 K, TlI NCs predominantly exhibit orthorhombic phase but have a minor contribution from cubic phase at $2\theta = 30^{o}$. With further increase in temperature to 443 K, the crystal structure of the NCs transforms to a predominant cubic phase, along with minor peaks from orthorhombic phase. A pure cubic phase, without any contribution from orthorhombic TlI, is observed at 483 K, and this cubic phase remains intact on further increase in temperature till 543 K (maximum temperature used in this study).

Then the NCs are cooled and the transition is monitored in the reverse direction from cubic to orthorhombic phase. But this reverse phase-transition has been observed to be a slow process with cubic phase dominating even after cooling the sample to 373 K. Surprisingly, the sample exhibits a minor contribution from cubic phase along with predominant orthorhombic phase, even after cooling the sample back to 303 K. It takes many hours at 303 K, before the sample completely converts back to a pure orthorhombic phase as shown by the XRD patterns in Figure 4a. One should note here that the full width at half maxima (FWHM) of XRD peaks and the lattice parameters remain unchanged before and after the reversible phase transition

of orthorhombic TlI NCs, suggesting that the average crystallite size remains intact during the temperature dependent phase transition study.

Phase transition in TlI NCs was further probed using DSC. Figure S5b shows a broad endotherm dip at ~446 K, corresponding to phase transition from orthorhombic to cubic phase in TlI NCs, and thus, commensurate with temperature dependent powder XRD results. This temperature dependent structural phase transition of TlI NCs has been schematically summarized in Figure 2c of the main manuscript.

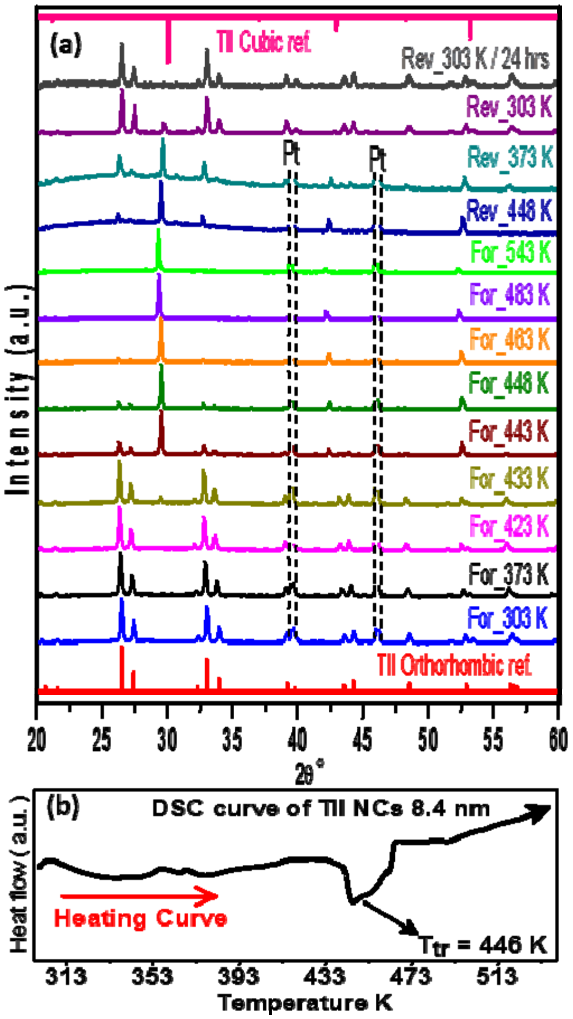

Figure S5: (a) Powder XRD patterns of TlI NCs with an average diameter of 8.4 nm, in the temperature range from 303 K to 543 K recorded both in forward (heating, represented as For_xyz K) and reverse (cooling, represented as Rev_xyz K) direction. (b) DSC plot showing broad endotherm dip at 446 K for orthorhombic to cubic phase transition, which is in agreement with high temperature XRD data.

Such structural phase transition can also change the optical properties. For example, orthorhombic phase of bulk TlI is known to exhibit an optical gap = 2.86 eV, whereas that of cubic phase is only 2.0 eV.[6] As expected, our TlI NCs also show similar structure dependent change in optical gap (see Figure S6). Photographs in the inset of Figure S6 show colour of colloidal TlI NCs is pale yellow at 30 °C (303 K, and remains so till temperature is as high as ~423 K), but it is reddish-orange at 220 °C (493 K).

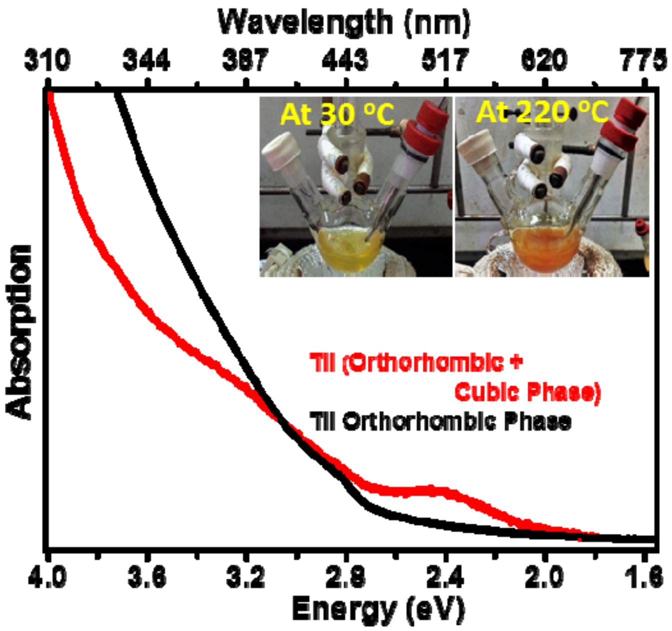

Figure S6: Influence of temperature dependent structural phase transition on optical properties of TlI NCs. Optical gap of TlI NCs are known to change from 2.86 eV for orthorhombic phase to 2.0 eV for cubic phase. Our colloidal TlI NCs also show a similar change in optical gap (UV-visible absorption spectra) and colour (photographs in the inset) for orthorhombic NCs at 30 ^{O}C to cubic NCs at 220 °C. For the sample at 220 °C, the hot colloidal dispersion was quickly transferred to a cuvette and the spectrum was collected within a minute at room temperature. In this process there is a small drop in temperature, which might be the reason for seeing some contribution from orthorhombic phase as well in the absorption spectrum after heating the sample at 220 °C.

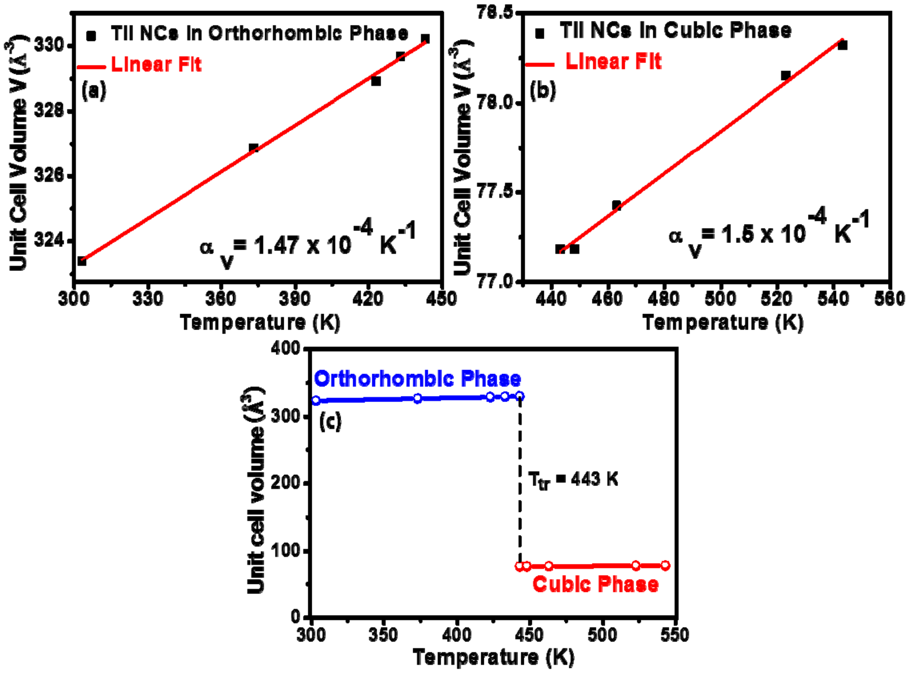

Figure S7: Unit cell volume of TlI NCs as a function of temperature for (a) orthorhombic phase (b) cubic phase. The calculated coefficient of volume expansion for orthorhombic phase in the temperature range of 303 K to 443 K is $\alpha_v = 1.47 \times 10^{-4}\ K^{-1}$, and that for cubic phase is $1.5 \times 10^{-4}\ K^{-1}$ in the range of 443 K to 543 K and**.** **(c)** Decrease in unit cell volume along phase transition from orthorhombic to cubic phase in TlI NCs. Please note that at the intermediate temperature of 443, a mixed phase is observed and unit cell volume of both phases were estimated from a single XRD pattern.

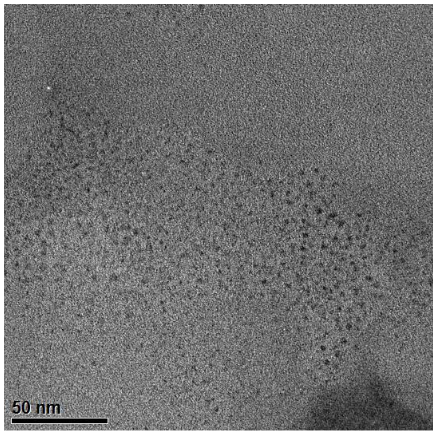

Figure S8: TEM image of 4 nm TlI NCs synthesized at 200 ^{o}C..

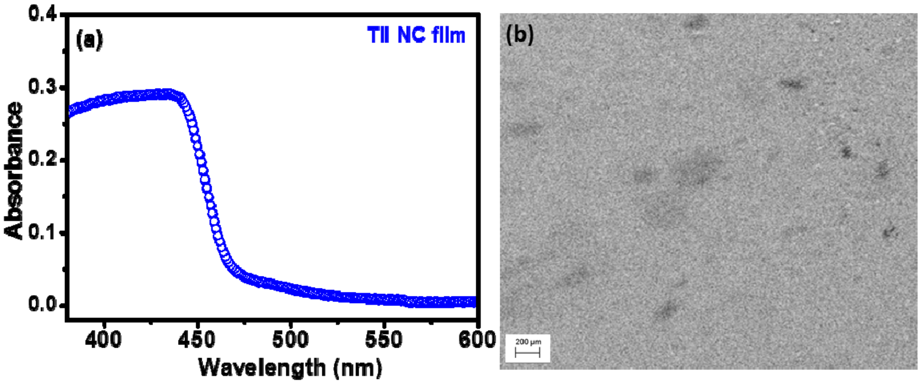

Figure S9: (a) UV-visible absorption spectrum of TlI NC film coated on polystyrene substrate. (b) SEM image of a region of the film showing uniform surface morphology, scale bar 200 μm.

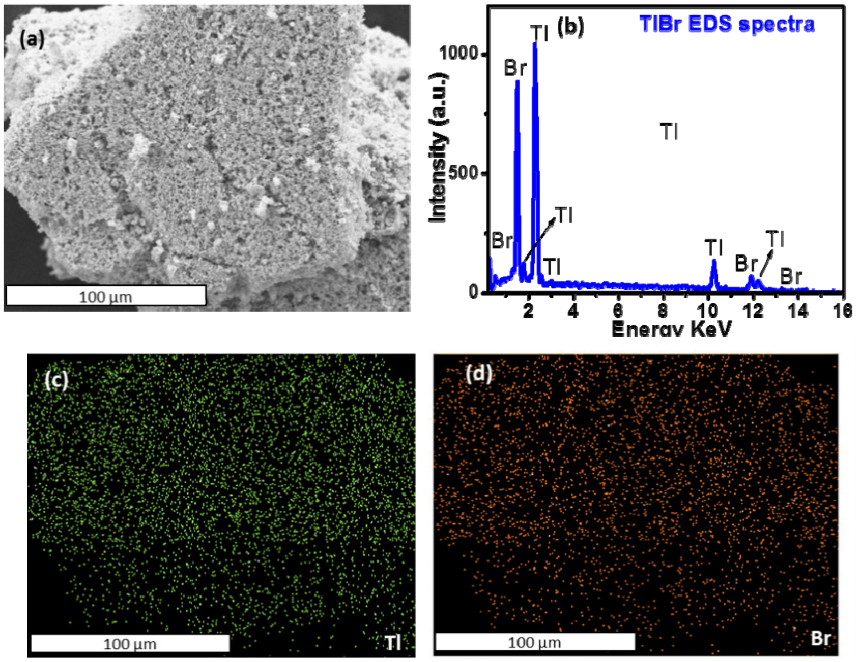

Figure S10: (a) SEM image of TlBr NCs. (b) EDS spectrum showing Tl:Br atomic ratio of 1:1. EDS mapping corresponding to the image in Figure S9a, for (c) Tl and (d) Br, showing homogeneous elemental distributions throughout the sample.

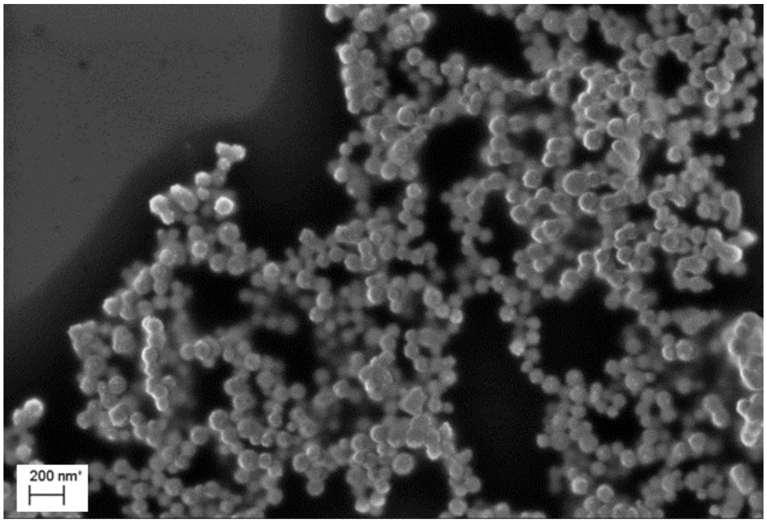

Figure S11: SEM image of TlBr NCs showing spherical morphology adapted by NCs.

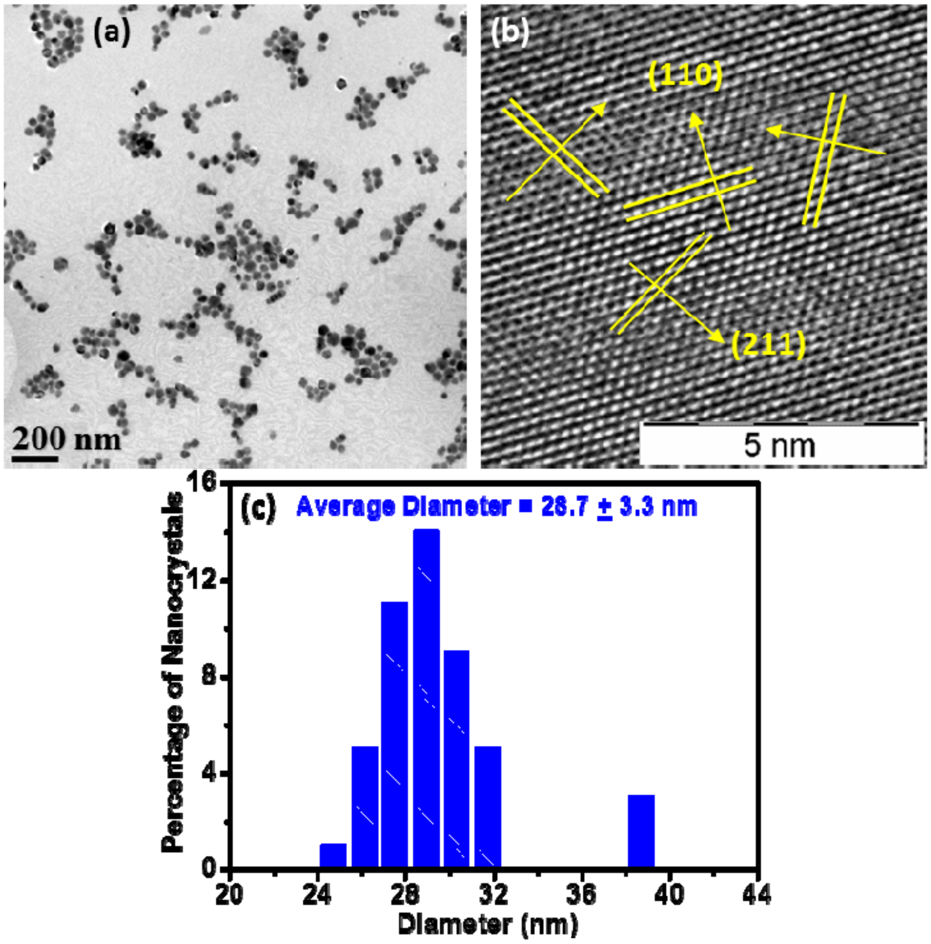

Figure S12: (a) TEM and (b) HRTEM image of TlBr NCs. Lattice fringes showing different interplanar distance are shown in the HRTEM image. (c) Size distribution histogram of TlBr NCs with average diameter 28.7 nm $\pm$ 3.3 nm.

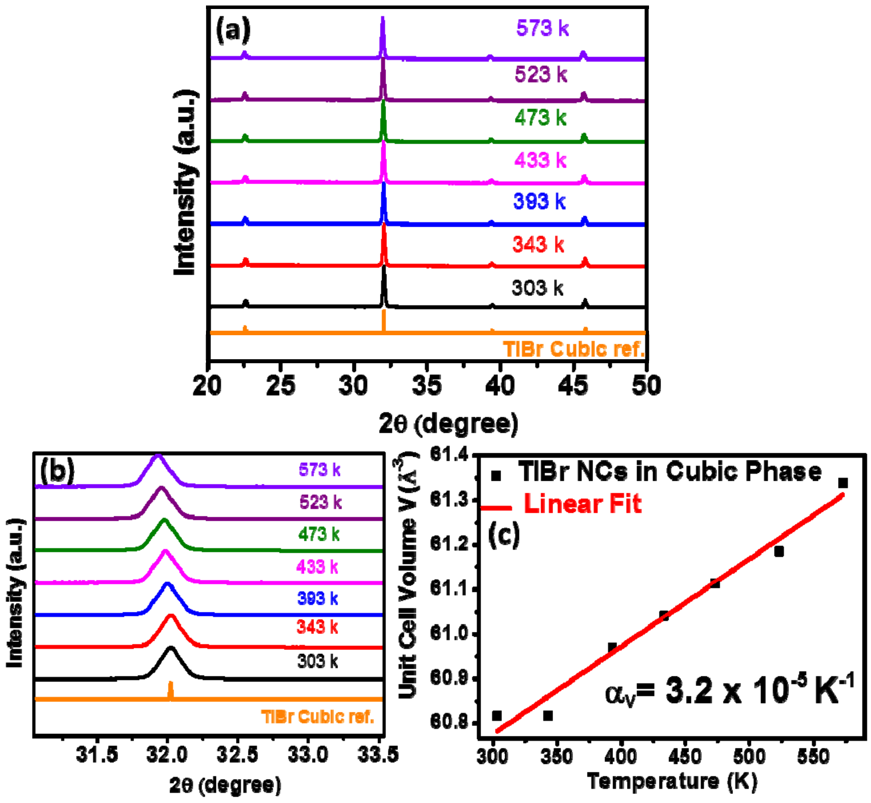

Figure S13: (a) Temperature dependent powder XRD pattern of TlBr NCs in the temperature range of 303 K to 573 K. No phase transition in this temperature range, however lattice expands with temperature. (b) Enlarged view of the highest intensity XRD peak of TlBr showing shift towards lower 2θ because of thermal expansion of unit cell with increasing temperature. (c) Linear behaviour of unit cell volume as a function of temperature yields coefficient of volume expansion $\alpha_v = 3.2 \times 10^{-5}$ K^{-1}.

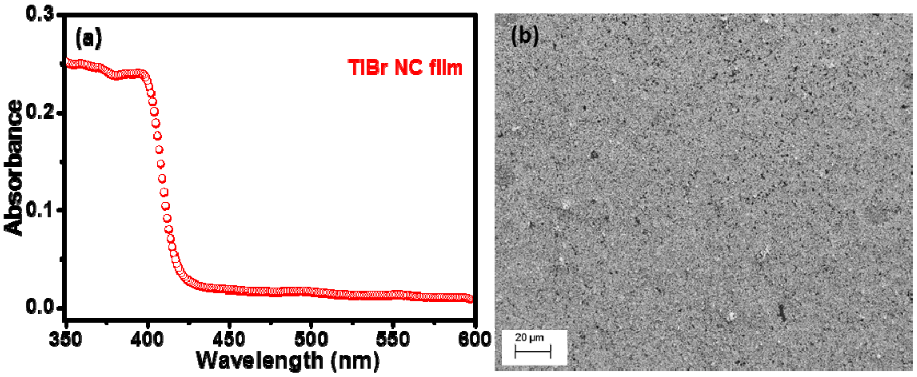

Figure S14: (a) UV-visible absorption spectrum of TlBr NC film coated on polystyrene substrate. (b) SEM image showing surface morphology of TlBr NC film, scale bar 20 µm.

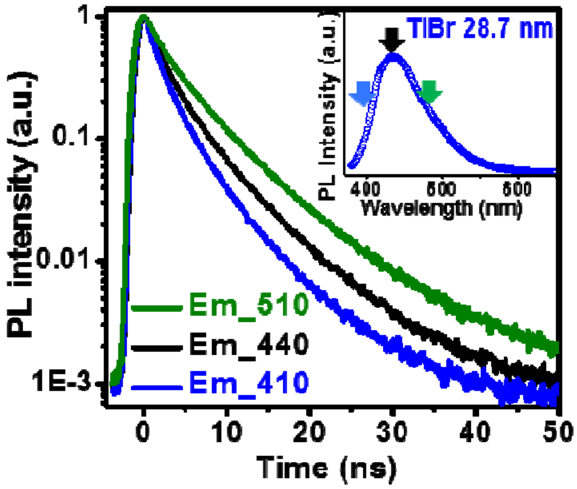

Figure S15: PL decay profiles of TlBr NCs (dispersed in toluene) at different emission wavelengths, after excitation at 340 nm.

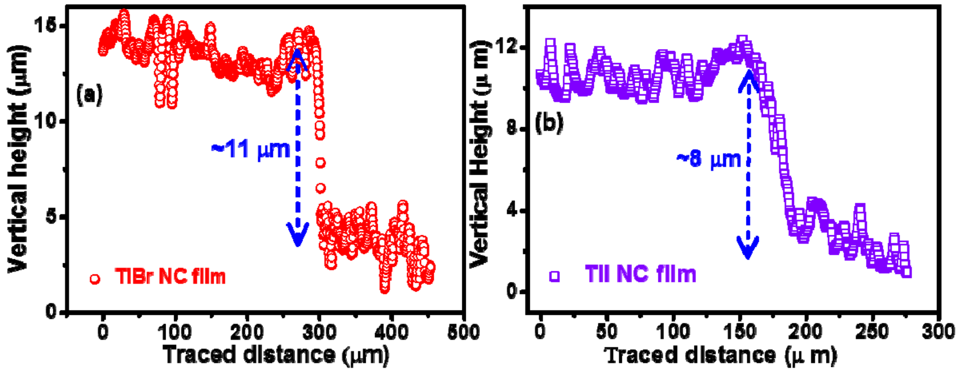

Figure S16: Optical surface profilometry scans of (a) TlBr NC, and (b) TlI NC film coated on polystyrene substrates. The obtained thickness of the film is ~11 μm for TlBr film and ~8 μm for TlI film.

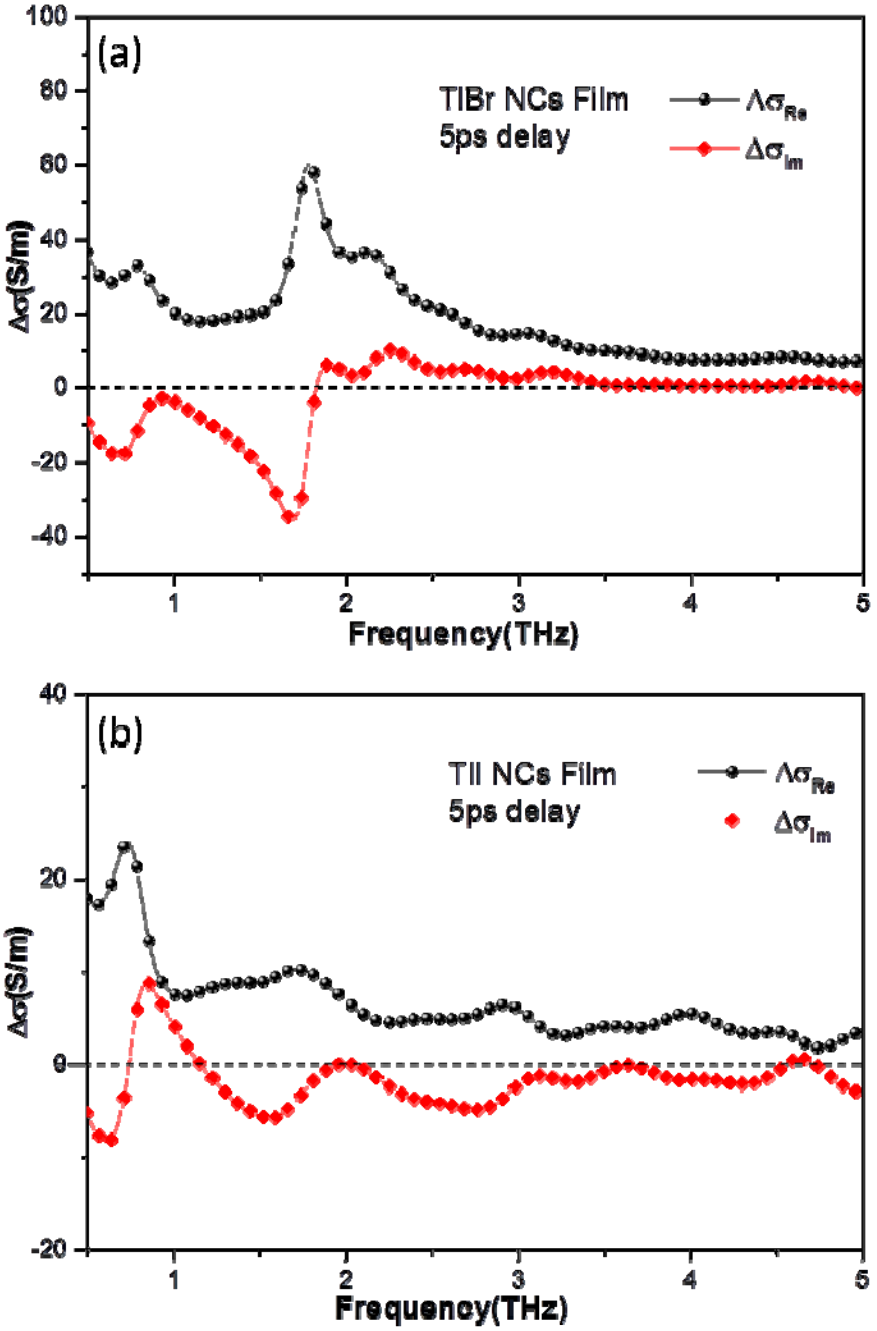

Figure S17: Real and imaginary conductivity spectra (pump induced) for (a) TlBr and (b) TlI at 27 and 23 μJ/cm^2 pump fluence respectively.

Table S1: The table shows reported effective and/ reduced masses of electron 'm_e' and hole 'm_h' in units of free electron mass 'm_o' for bulk TlI and TlBr. Using these reported excitonic parameters we calculate Bohr diameter using well known equation $\frac{\hbar^2\mathcal{E}}{\mu e^2}$, where μ is reduced mass, e is charge of electron and $\mathcal{E}$ is dielectric constant. The dielectric constant values were taken from literature ($\mathcal{E}$ = 30.6 for bulk TlBr and 21.2 for bulk TlI at 25 ^{o}C). In some cases Bohr diameter are directly reported from literature without requiring any further calculation, and are denoted by '#' sign.

Sample	effective mass of electron (m_e)	effective mass of hole (m_h)	reduced mass (μ) m_o	method	excitonic Bohr diameter (nm)	ref.
**TlI**			0.25	Magneto-Optical absorption	**8.8**	[7]
				Absorbance of microcrystals	**(10 – 12)$^{\#}$**	[8]
**TlBr**			0.23	Magneto-Optical absorption	**13.9**	[9]
	0.28 m_o	0.72 m_o	0.20	Magneto-Optical absorption	**16**	[10]
	0.55 m_o	0.52 m_o	0.26	Cyclotron effective mass	**12.3**	[11]
			0.72	Absorbance of microcrystals	**4.5**	[12]
	0.52 m_o	0.66 m_o	0.29	High density excitation luminescence and theoretical calculation	**11**	[13]
	0.2 m_o	0.4 m_o	0.13	Relativistic KKR band structure	**24.6**	[14]
	0.51 m_o	0.98 m_o	0.33	DFT band structure	**9.7**	[15]

Table S2: Tri-exponential decay fitted parameters of TlI NCs at different emission wavelength as shown in table. 'τ_i' is the component of lifetime for the ith process and 'a_i' is its coefficient of contribution.

emission wavelength	τ_1 (ns)	τ_2 (ns)	τ_3 (ns)	a_1	a_2	a_3
460 nm	1.1	3.9	11.7	0.63	0.32	0.04
478 nm	1.2	4.2	12.6	0.54	0.40	0.05
540 nm	1.4	4.6	12.7	0.44	0.47	0.07

Table S3: Details of parameters obtained from fitting the THz transient data to multi-exponential decay function (equation 2) at different excitation fluences for TlBr.

TlBr-NC film on polystyrene	29 µJ/cm^2	24 µJ/cm^2	17 µJ/cm^2	11 µJ/cm^2	7 µJ/cm^2
τ_1	51.2 ± 0.2	44.5 ± 0.2	40.5 ± 0.2	22.0 ± 0.2	31.8 ± 0.3
a_1	0.405 ±0.002	0.481 ± 0.003	0.523 ±0.002	0.534 ± 0.002	0.586 ± 0.004
τ_2	254 ± 3	240 ± 2	227 ± 3	172 ± 2	156 ± 3
a_2	0.286 ± 0.001	0.223± 0.004	0.251 ± 0.001	0.313 ± 0.001	0.212 ± 0.003
τ_3	1345 ± 9	1133 ± 11	1269 ± 13	1664 ± 23	1655 ± 24
a_3	0.269 ± 0.002	0.282 ± 0.002	0.208 ± 0.002	0.132 ± 0.001	0.148 ± 0.001
$\langle\tau\rangle$	1112	953	1020	1315	1386

Table S4: Details of parameters obtained from fitting the THz transient data to multi-exponential decay function at different excitation fluences for TlI.

TlI-NC film on polystyrene	27 µJ/cm^2	23 µJ/cm^2	17 µJ/cm^2	11 µJ/cm^2	5 µJ/cm^2
τ_1	32.2 ± 0.1	35.4 ± 0.1	24.8 ± 0.4	22.0 ± 0.5	71.4 ± 0.2
a_1	0.641 ± 0.002	0.679 ± 0.002	0.371 ± 0.004	0.443 ± 0.003	0.831 ± 0.002
τ_2	137.6 ± 0.5	146.7 ± 0.7	93.9 ± 0.4	136.9 ±0.5	
a_2	0.353 ± 0.002	0.303 ± 0.002	0.643 ± 0.005	0.536 ±0.002	
$\langle\tau\rangle$	106.2	107.6	84.8	123.4	71.4

References:

1.M. C. Beard, G. M. Turner and C. A. Schmuttenmaer, *J. Phys. Chem. B*, 2002, **106**, 7146-7159.
2. R. Ulbricht, E. Hendry, J. Shan, T. F. Heinz and M. Bonn, *Rev. Mod. Phys.*, 2011, **83**, 543-586.
3. J. B. Baxter and G. W. Guglietta, *Anal. Chem.*, 2011, **83**, 4342-4368.
4. B. Clough, J. Dai and X. C. Zhang, *Mater. Today*, 2012, **15**, 50-58.
5. K. Iwaszczuk, D. G. Cooke, M. Fujiwara, H. Hashimoto and P. U. Jepsen, *Opt. Express*, 2009, **17**, 21969-21976.
6. K. Heidrich, W. Staude, J. Treusch and H. Overhof, *Solid State Commun.,* 1975, **16**, 1043-1045.
7. A. Fujii, K. Takiyama, J. i. Nakahara and K. Kobayashi, *J. Phys. Soc. Jpn.*, 1977, **42**, 525-528.
7. V. F. Agekyan, I. Akai and T. Karasawa, *Phys. Solid State*, 2003, **45**, 1170-1176.
8. S. Kurita and K. Kobayashi, *J. Phys. Soc. Jpn.*, 1971, **30**, 1645-1653.
9. R. Z. Bachrach and F. C. Brown, *Phys. Rev. B*, 1970, **1**, 818-831.
10. J. W. Hodby, G. T. Jenkin, K. Kobayashi and H. Tamura, *Solid State Commun.*, 1972, **10**, 1017-1020.
11. V. F. Agekyan and Y. A. Stepanov, *Phys. Solid State*, 2001, **43**, 763-765.
12. K. Takiyama, J. Nakahara, H. Takenaka, K. Kobayashi, T. Fujita and A. Fujii, *J. Phys. Soc. Jpn.*, 1996, **65**, 307-311.
13. K. Heidrich, W. Staude, J. Treusch and H. Overhof, *Phys. Rev. Lett.*, 1974, **33**, 1220-1223.
14. C. R. Leão and V. Lordi, *Phys. Rev. Lett.*, 2012, **108**, 246604.
